# Dishevelled-1 DIX and PDZ domain lysine residues regulate oncogenic Wnt signaling

**DOI:** 10.18632/oncotarget.28089

**Published:** 2021-10-26

**Authors:** Monica Sharma, Isabel Castro-Piedras, Fahmida Rasha, Sabarish Ramachandran, Souad R. Sennoune, Kathryn Furr, Sharilyn Almodovar, Vadivel Ganapathy, Matthew B. Grisham, Rakhshanda Layeequr Rahman, Kevin Pruitt

**Affiliations:** ^1^Immunology and Molecular Microbiology, Texas Tech University Health Sciences Center, Lubbock, TX, USA; ^2^Cell Biology and Biochemistry, Texas Tech University Health Sciences Center, Lubbock, TX, USA; ^3^Department of Surgery, Texas Tech University Health Sciences Center, Lubbock, TX, USA

**Keywords:** dishevelled (DVL), post-translational modification, lysine residue, gene expression, chromatin immunoprecipitation (ChIP)

## Abstract

DVL proteins are central mediators of the Wnt pathway and relay complex input signals into different branches of the Wnt signaling network. However, molecular mechanism(s) that regulate DVL-mediated relay of Wnt signals still remains unclear. Here, for the first time, we elucidate the functional significance of three DVL-1 lysines (K/Lys) which are subject to post-translational acetylation. We demonstrate that K34 Lys residue in the DIX domain regulates subcellular localization of β-catenin, thereby influencing downstream Wnt target gene expression. Additionally, we show that K69 (DIX domain) and K285 (PDZ domain) regulate binding of DVL-1 to Wnt target gene promoters and modulate expression of Wnt target genes including *CMYC*, *OCT4*, *NANOG*, and *CCND1*, in cell line models and xenograft tumors. Finally, we report that conserved DVL-1 lysines modulate various oncogenic functions such as cell migration, proliferation, cell-cycle progression, 3D-spheroid formation and *in-vivo* tumor growth in breast cancer models. Collectively, these findings highlight the importance of DVL-1 domain-specific lysines which were recently shown to be acetylated and characterize their influence on Wnt signaling. These site-specific modifications may be subject to regulation by therapeutics already in clinical use (lysine deacetylase inhibitors such as Panobinostat and Vorinostat) or may possibly have prognostic utility in translational efforts that seek to modulate dysfunctional Wnt signaling.

## INTRODUCTION

Wnt signaling is integral for normal development and tissue homeostasis. With 19 Wnt ligands, 10 Frizzled receptors and 3 Dishevelled (DVL) proteins participating in signaling, the amount of information relayed is enormous. The canonical or β-catenin dependent branch of Wnt signaling relays information through secreted Wnt ligands, transmembrane Frizzled receptors, low-density lipoprotein receptor related protein 5/6 (LRP5/6), DVL proteins, and active (non-phosphorylated) β-catenin to initiate transcription of Wnt target genes. Canonical signals are crucial for diverse cell behaviors, including cell fate, cell survival, proliferation, and stem cell renewal. The non-canonical branch, while less characterized, regulates equally important cellular functions such as cell organization, adhesion and polarity. The non-canonical branch can be further classified into planar cell polarity (PCP) pathway and Wnt/Ca^2+^ pathway [1, 2].

The activation of Wnt signaling is tightly regulated by post-translational modifications (PTM) of several pathway components, with phosphorylation of β-catenin being the most intensely investigated. PTMs are highly dynamic and reversible and several PTMs have been identified on key Wnt components including phosphorylation, acetylation, methylation, and ubiquitination which influence the ability of targets to relay signaling and alter cellular responses [3, 4]. DVL integrates and transmits complex Wnt signals, yet how it conducts this symphony of activity remains unclear. Regulation by various PTMs, however, have been shown to play a critical role specifying how the molecular signals are routed. Some of the well-studied PTMs of DVL include phosphorylation [5–12], ubiquitination [13–16], and methylation [17]. We recently reported post-translational acetylation on 12 key DVL-1 lysine residues as a novel PTM, present in the highly conserved DIX, PDZ and DEP domains [18]. Prior to our recent report, only a few components of the Wnt pathway such as β-catenin, and TCF had been shown to be acetylated [19]. Here, we characterize the functional significance of specific DVL-1 lysines which are subject to post-translational acetylation. While studies have highlighted ubiquitination-mediated regulation on lysines of DVL-2 [14, 15, 20, 21] and DVL-3 [22, 23], there are no previous studies investigating the role of DVL-1 lysines that undergo acetylation in two important DVL domains with respect to Wnt signaling or oncogenic cellular functions. Our results indicate that unlike K69 and K285, K34 does not control nuclear localization of DVL-1 across multiple cell line models. For the first time we demonstrate that all 3 DVL-1 residues (K34, K69, K285) differentially regulate canonical Wnt signaling [3]. However, the K34 site is particularly potent in regulating active (non-phosphorylated) β-catenin levels and total β-catenin sub-cellular localization. Moreover, each of these individual lysine residues, which are subject to post-translational acetylation, controls the expression of key Wnt target genes such as *AXIN2, SOX17, NANOG, OCT4*, and *CCND1*.

Very intriguingly, while the K69 and K285 residues not only regulate DVL-1 nuclear translocation, they also regulate gene expression by modulating DVL-1 binding to Wnt target gene promoters in different breast cancer models, yet again indicating a more expansive role of DVL beyond the cytoplasm [24–26]. Moreover, using *in vitro* and *in vivo* cell-biology based assays, we show for the first time that specific lysines regulate cell migration, proliferation, cell-cycle progression, and xenograft tumor growth. Collectively, these findings demonstrate that three conserved key lysines which undergo acetylation and deacetylation cycles, act as a potent modulator of the Wnt pathway. These findings further suggest that lysine residues which undergo druggable and reversible PTMs could potentially coordinate oncogenic Wnt signaling networks.

## RESULTS

### Higher protein expression of DVL-1 promotes tumor growth in triple-negative breast cancer

**Figure 1:**
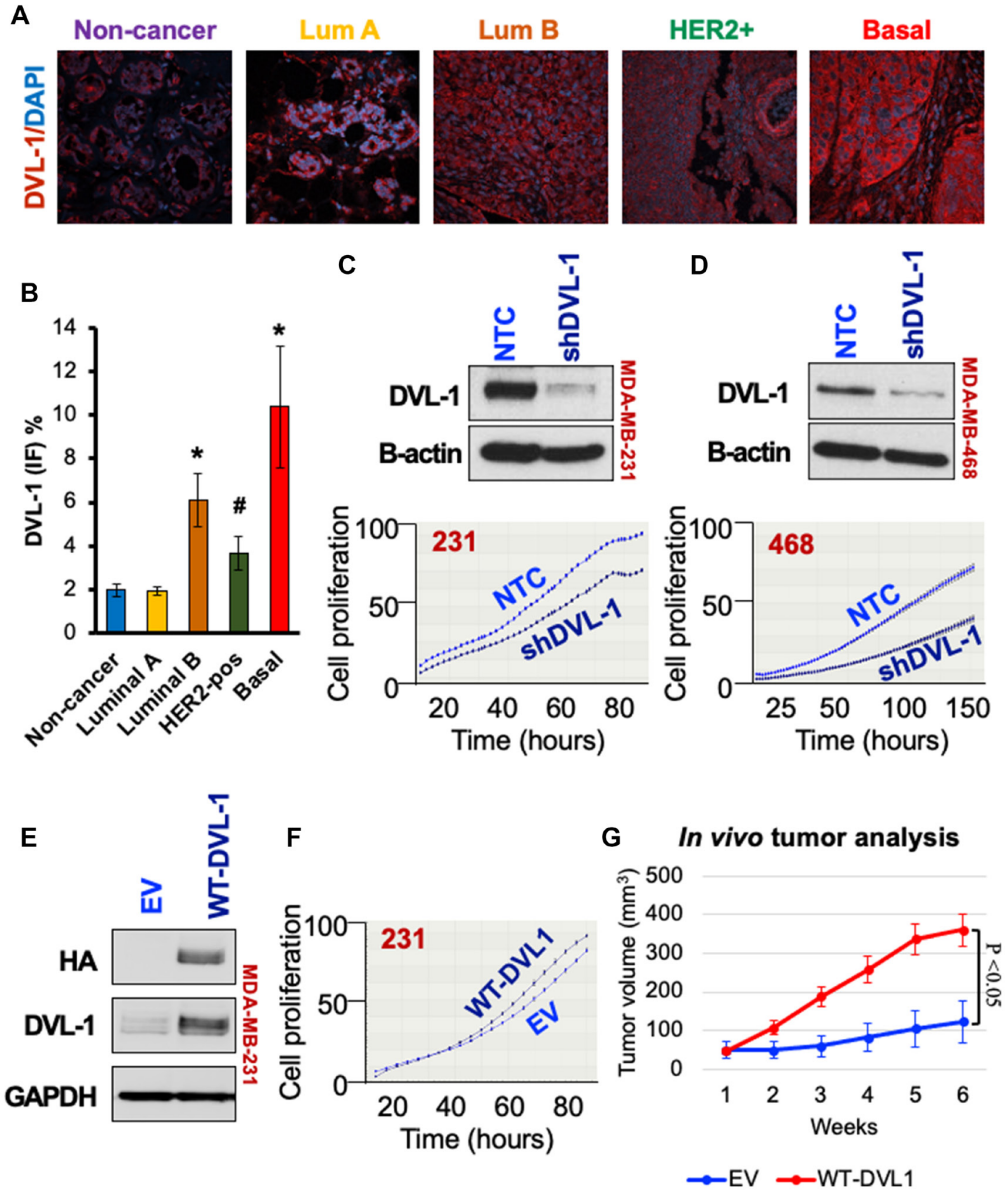
High DVL-1 expression promotes cell proliferation and xenograft tumor growth in triple-negative breast cancer. (**A**) Merge of immunofluorescence staining for DVL-1 (red) and nucleus (blue) in different primary human breast cancer tissues (including non-cancer adjacent tissue, luminal A, luminal B, HER2+, and basal/TNBC). (**B**) Graphical representation of average DVL-1 staining from multiple breast cancer patients in various subgroups (*n* ≥ 3; ^*^ *p* < 0.05; ^#^ *p* < 0.1). Western blot for DVL-1 protein expression in NTC and shDVL1 knockdown in (**C**) MDA-MB-231 and (**D**) MDA-MB-468 cells. Graph below represents cell proliferation rate quantified in real time by live cell imaging using the IncuCyte ZOOM (Essen Biosciences). Images were collected every 2 hours for 150 hours. (**E**) Western blot for DVL-1 expression in empty vector (EV) versus wild-type DVL1 gain-of-function in MDA-MB-231. (**F**) Cell proliferation in EV versus WT-DVL1 cells quantified in real time by live cell imaging, where images were collected every 2 hours for 80 hours. (**G**) Mean tumor volume of EV and WT-DVL1 MDA-MB-231 cells subcutaneously injected in mammary fat pad of immunocompromised mice (*n* = 5 per group, *p* < 0.05). Please refer to Supplementary Figure 2 for additional information for western blot panels.

We had previously reported that DVL-1 protein expression was higher in triple-negative breast cancer cell (TNBC) line models [18]. To further assess the levels of DVL-1 protein expression in primary breast cancers, we performed immunofluorescence staining on resected tumors from patients with known molecular subtypes. We observed that DVL-1 protein levels were significantly higher in luminal B and HER2 amplified tumors, but the highest expression was in triple-negative breast cancers compared to non-cancer tissues (Figure 1A–1B). We next investigated the impact of DVL-1 on cancer hallmarks in TNBC models. To study its relevance *in-vitro*, we generated stable DVL-1 knock-down cell lines. Using short hairpin RNAs (shRNA) that targets coding region of DVL-1 mRNA vs. a non-targeting control (NTC), we developed two cell lines that have reduced expression of DVL-1 (Figure 1C–1D) in MDA-MB-231 and MDA-MB-468. Proliferation analyses revealed that knockdown of DVL-1 (shDVL-1) resulted in reduced proliferation compared to non-targeted control in both TNBC cell lines. In contrast, proliferation increased in MDA-MB-231 cells stably over-expressing wild-type DVL1 (WT) compared to empty-vector control (EV) cells (Figure 1E–1F). Given these findings, we extended our analysis to assess the impact of DVL-1 gain-of-function *in vivo*. We performed xenograft tumor experiments by injecting MDA-MB-231 stably expressing EV vs. WT-DVL1 in the mammary fat pad of athymic nude mice. We observed that WT-DVL1 significantly increased MDA-MB-231 growth in immunocompromised mice (Figure 1G, Supplementary Figure 1A). Collectively, these results demonstrate that DVL-1 enhances tumor growth, both *in-vitro* and *in-vivo* in TNBC models.

### Highly conserved lysines in the DIX and PDZ domains which are subject to post-translational acetylation are critical for DVL-1 subcellular localization and its protein binding capacity

The structural domains of DVL proteins namely, the DIX, PDZ and DEP domain play an integral role in channeling Wnt signals to canonical and non-canonical branches, to promote either cell proliferation or migration/polarity, respectively. DVL domains harbor lysine residues which are highly conserved across different species [18] which might be regulated by several post-translational modifications (Supplementary Figure 3A–3B, Table 1). Previously, we had shown that 12 highly conserved lysine residues on DVL-1 undergo post-translational acetylation in varying oxygen tension and deacetylase inhibition in TNBC cells. We also identified that K69 and K285 residues in the DIX and PDZ domains respectively, are critical for sub-cellular localization of DVL-1 [18]. Here, we extend our study to patient-derived xenograft (PDX) TNBC models with active Wnt signaling. In this PDX model we have identified highly conserved lysine residues such as K34 within the DIX domain of DVL-1 which consistently undergoes post-translational acetylation in various models we have tested so far (Supplementary Figures 3C–3D and 4).

**Table 1:** K34, K69 and K285 residues on DVL-1 are subject to multiple PTMs

Residue	PTM Modification	Mod Pred Score	Confidence
K34	Acetylation	0.61	Low
	SUMOylation	0.63	Low
	Methylation	0.57	Low
	Ubiquitination	0.68	Medium
K34R	Proteolytic cleavage	0.73	Medium
K34Q	Not modified	–	–
K69	Ubiquitination	0.58	Low
	SUMOylation	0.68	Low
K69R	Not modified	–	–
K69Q	Not modified	–	–
K285	Acetylation	0.57	Low
	Methylation	0.57	Low
	Ubiquitination	0.65	Low
K285R	Methylation	0.75	Medium
	Proteolytic cleavage	0.52	Low
K285Q	Not modified	–	–

**Figure 2:**
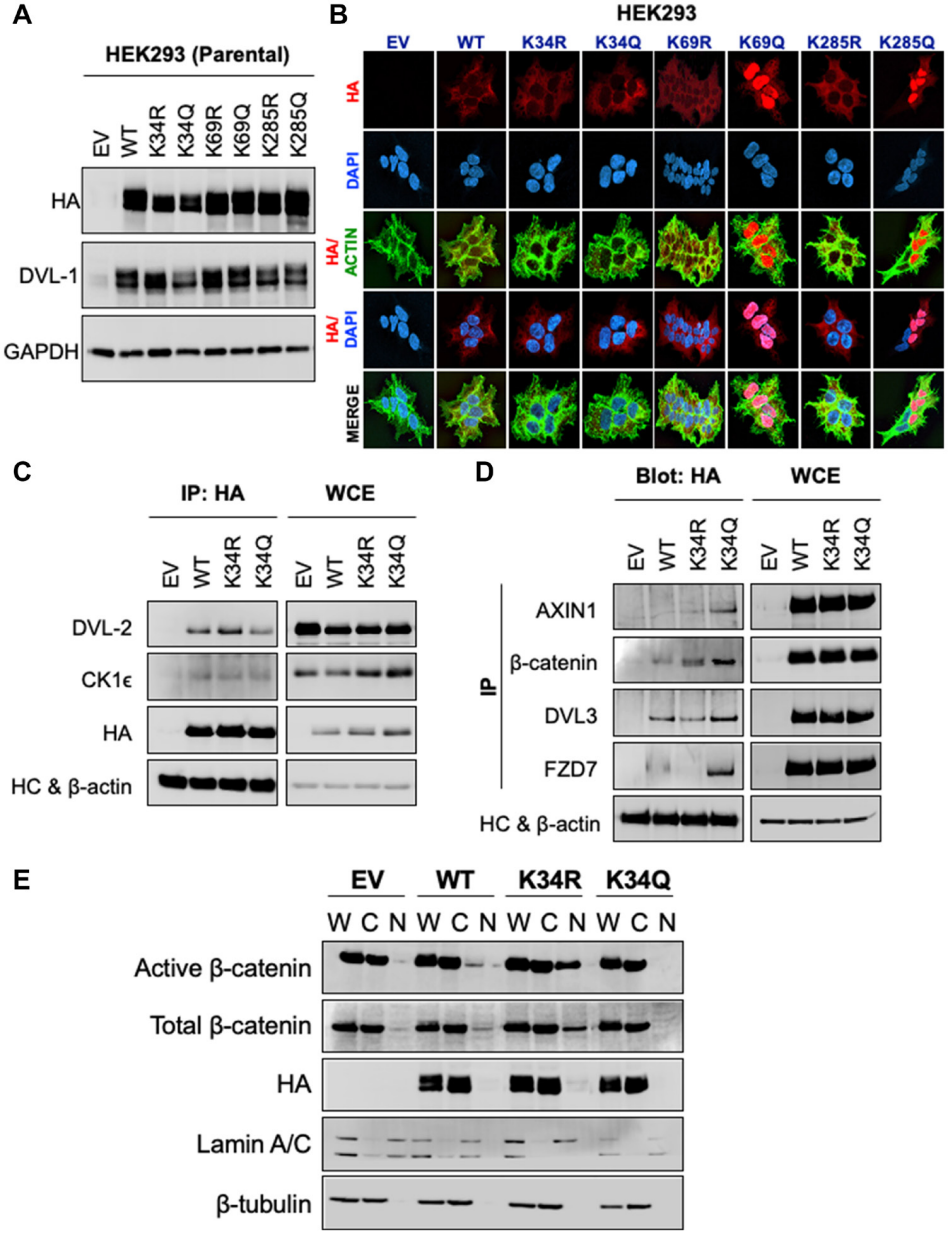
K34 residue regulates DVL-1 protein-protein interaction and entry of β-catenin into the nucleus, while K69 and K285 are critical for DVL-1 subcellular localization. (**A**) Western blot analysis of lysates from HEK293 cells stably expressing empty vector (EV), N-terminal HA-epitope tagged DVL-1 wild type (WT), HA-tagged deacetylation mutants (K to R), HA-tagged acetylation mutants (K to Q) on highly conserved lysine residues namely, K34, K69 and K285, probed with antibody as indicated. Also see Supplementary Figure 6 for additional information for western blot panels. (**B**) Immunofluorescence staining of empty vector (EV), N-HA-tagged DVL-1 (WT), K34R mutant (N-HA-K34R), K34Q mutant (N-HA-K34Q), K69R mutant (N-HA-K69R), K69Q mutant (N-HA-K69Q), K285R mutant (N-HA-K285R), K285Q mutant (N-HA-K285Q), proteins in stably expressing HEK293 cells. Merge of N-HA-DVL-1 (red) and nuclear staining (blue) proteins is shown as HA/DAPI for each of the mutant. Merge of actin (green) and N-HA-DVL-1 (red) proteins is shown as HA/Actin for each of the mutant. For co-immunoprecipitation (Co-IP) experiments, HEK293 cells stably expressing DVL-1 mutants at K34 residues were used where we immunoprecipitated using antibodies against (**C**) HA-epitope and blotted for binding partners such as DVL-2 and casein kinase 1 (CK1e) versus (**D**) a reciprocal Co-IP was performed to pull down different binding partners such as Axin1, DVL3, β-catenin, FZD7 and blotted for HA-tag. Empty vector (EV) was used as a negative control, and whole cell extracts (WCE) as a positive control. IgG heavy chain (HC) was blotted for as a control for equal antibody loading for immunoprecipitation. β-actin was included as a loading control for WCE. See Supplementary Figure 7 for additional information for western blot panels. (**E**) Whole (W), cytoplasmic (C) and nuclear (N) extracts from HEK293 cells stably expressing EV, WT-DVL1, K34R, and K34Q were analyzed using Western blots. The blots were probed with antibodies against active (non-phosphorylated) β-catenin, total β-catenin, and HA-tag. Lamin was used as a control for nuclear extract and β-tubulin was used as a control for cytosolic proteins. See Supplementary Figure 8 for additional information for western blot panels.

Given the critical role of DVL as a central mediator of Wnt signaling, we next addressed the key unknowns of the functional significance of these three different lysines with respect to Wnt signaling impacting broad cellular functions. To understand the impact of these conserved lysines, we substituted K for arginine (R) or glutamine (Q). We chose R and Q for substitutions to conserve the length of the side chain. They were also chosen given the differences in charge. Since lysine acetylation neutralizes the positive charge we substituted K with a neutrally-charged glutamine (K>Q). Since the deacetylated lysine retains the positive charge, we also substituted the positively charged lysine for the positively charged arginine (K>R). While these substitutions come with caveats, they provide a good first step at assessing the functional significance of DVL-1 lysines that are subject to acetylation/deacetylation cycles [3]. Hence, to probe the functional significance of DVL-1 lysines that are subject to acetylation, and with respect to Wnt signaling, we used HEK293 and HEK293Trex DVL-TKO cells, where the latter model has DVL1-3 deleted via CRISPR-Cas9. In both lines, we created isogenic clones stably expressing empty vector (EV), HA-tagged DVL-1 (WT-DVL1), and HA-tagged K>R and HA-tagged K>Q mutants at K34, K69, and K285 residues. Comparable protein expression of N-HA-hDVL1 constructs was confirmed for WT and all six point mutants in HEK293 (Figure 2A) and HEK293Trex DVL-TKO cells (Supplementary Figure 5A). Similar to our previous findings, we observed that K>Q substitution of K69 and K285, but not K34, promote nuclear over cytosolic localization of DVL-1 in HEK293 and HEK293Trex DVL-TKO cells (Figure 2B and Supplementary Figure 5B), suggesting that contribution of these residues may be relatively consistent across cellular contexts. Furthermore, unlike the punctae formation with DVL2-K68 acetylation as shown in Shen et al. [27], we did not observe any difference in patterns of punctae in either R or Q mutants for DVL-1 in our study. More investigation is needed to determine whether the difference in punctae formation is due to an intrinsic difference in the impact of PTMs on regulation of DVL paralogs (DVL-1 vs. DVL-2) or a difference in the cellular context given the different cell lines used.

An important function of DVL is to associate with binding partners and relay information into WNT signaling branches; therefore, we next determined the influence of specific lysine residues, which are subject to acetylation, in modulating interaction of DVL-1 with its protein binding partners. Key Wnt signaling regulators such as Axin1, b-catenin, Frizzled, casein kinase 1 (CK1e), and other DVL proteins (DVL-2 and DVL-3) interact with DVL-1 and mediate constitutive WNT signaling [2, 28]. To understand the functional consequences of the DVL-1 K34R on protein binding, we performed co-immunoprecipitation studies using K34R and K34Q, both of which localize to the cytoplasm. Wild-type DVL-1 (WT), K34R or K34Q were immunoprecipitated using HA antibody, and the co-precipitation of aforementioned interacting proteins was assessed. Consistently DVL2/3, CK1e, and b-catenin were co-precipitated with WT-DVL1 and K34 mutants (Figure 2C–2D). However, the K34Q showed a significant increase in binding to Axin1, DVL3, b-catenin, and FZD7, compared to WT-DVL1 and K>R point mutants. We, however, did not observe any significant changes in binding with K>R and K>Q point mutants at K69 or K285 residues with respect to WT-DVL1 (Supplementary Figure 9). This suggests that lysine K34 is important for DVL-1 interaction with various critical Wnt pathway regulators. Overall, these experiments provided critical insights: (a) K34 is critical for DVL-1 protein-scaffolding property for specific binding partners such as Axin1, DVL3, FZD7 and b-catenin, while (b) K69 and K285 modulate DVL-1 nuclear localization in HEK293 cells, suggesting that this regulatory switch might be critical across broader biological contexts.

### Conserved K34 residue of DVL-1 impairs nuclear localization of β-catenin and alters WNT target gene expression in multiple cell lines

β-catenin is tightly regulated at the levels of protein stability, cellular localization and transcriptional activity. Accumulation of nuclear β-catenin results in active Wnt signaling and promotes transcription of many target genes. We next determined the relative steady-state levels of active (non-phosphorylated) β-catenin levels with respect to K34 mutants. We found that active (non-phosphorylated) and total β-catenin levels were appreciably higher in the nuclear compartment in the K34R compared to WT-DVL1. However, in the presence of K34Q, the levels of active and total β-catenin were considerably lower in the nucleus (Figure 2E and Supplementary Figures 1B, 10A and 11).

**Figure 3:**
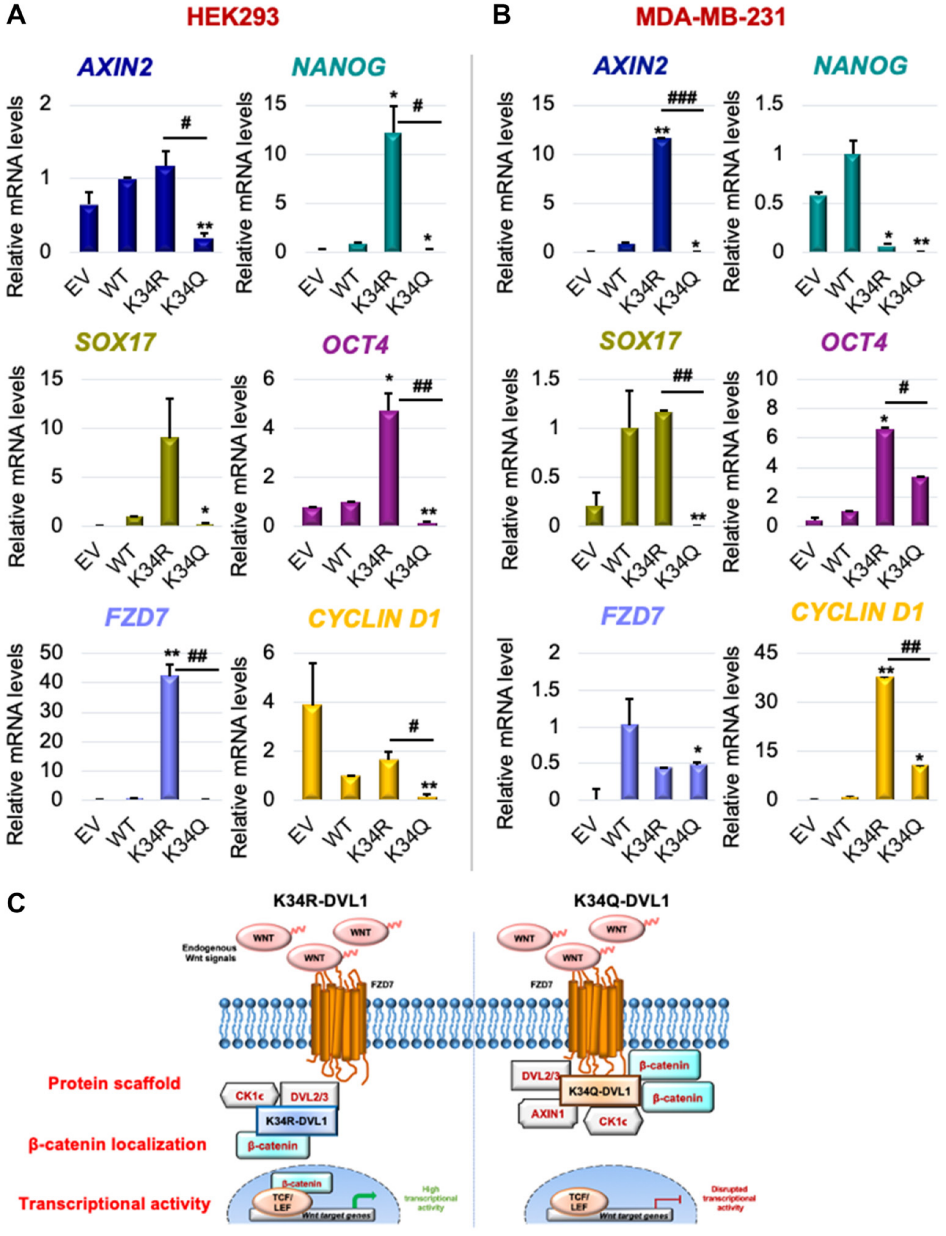
Conserved K34 residue on DVL-1 regulates canonical WNT target gene expression in various cellular models. mRNA expression of WNT target genes such as *AXIN2*, *NANOG*, *SOX17,* *OCT4*, *FZD7*, and *CYCLIND1* was determined by qRT-PCR (normalized to B2M as control) in (**A**) HEK293 and (**B**) MDA-MB-231 cells stably expressing EV, WT-DVL1, K34R, and K34Q mutants. The sign “^*^” represents significant change in gene expression between WT and mutants, while “^#^” represents significant change between R and Q mutants. All results are expressed as mean ± SEM and considered significant at ^*/#^ *p* < 0.05, ^**/##^ *p* < 0.01 and ^***/###^ *p* < 0.001. (**C**) Schematic representation of the functional significance of DVL1-K34 residue with respect of Wnt signaling. Mutation on highly conserved K34 residue impacts DVL-1 protein scaffolding property, entry of β-catenin into the nucleus, thereby downregulating canonical WNT target gene expression.

Since DVL-1 K34 mutants influenced relative levels and subcellular localization of b-catenin, we further assessed its influence on downstream Wnt signaling. We performed mRNA expression analysis for a few Wnt target genes and other DVL target genes we identified in different cell line models. First, real-time RT-PCR analysis performed in HEK293 cells showed that the K34Q caused a significant reduction in the mRNA levels of several different Wnt target genes (Figure 3A and Supplementary Figure 10B). We also analyzed MDA-MB-231 stably expressing DVL-1 mutants (Figure 3B). We observed very similar trends with changes in mRNA expression of Wnt target genes, where DVL-1 K34Q caused a significant reduction in the mRNA levels of several different Wnt target genes including *AXIN2, SOX17, NANOG, OCT4*, and *CYCLIND1* in MDA-MB-231 cells compared to DVL-1 WT and K34R (Please refer to Supplementary Figure 12 for Wnt3A stimulation). Several reports have demonstrated these genes are regulated by DVL proteins and critical for cancer growth [29–31]. Interestingly, with the K34R, which induces higher levels of nuclear β-catenin, we observe a significant increase in expression of the pluripotency regulators such as *SOX17*, *NANOG*, *OCT4* in multiple cell lines, genes that are essential for maintaining human embryonic stem cells [32]. Moreover, previously we had shown that DVL-1 forms a complex with c-Jun and enriches at FZD7 promoter in breast cancer cells [33]. Here, we show that DVL-1 K34R significantly upregulates mRNA expression of FZD7, whereas K34Q results in a reduction in FZD7 mRNA levels, thereby suggesting that DVL-1 post-translational acetylation regulates transcription of FZD7, a major Wnt ligand receptor.

Collectively, these results suggest that PTMs of the conserved K34 residue likely regulate protein scaffolding properties of DVL-1, impacting subcellular localization of β-catenin and influencing downstream Wnt target gene expression. This represents the first demonstration of K34 of DVL-1 participating in a novel regulatory mechanism of a critical component of Wnt signaling pathway (Figure 3C).

### Lysines K69 and K285 influence DVL-1 promoter binding and regulation of multiple WNT target genes

**Figure 4:**
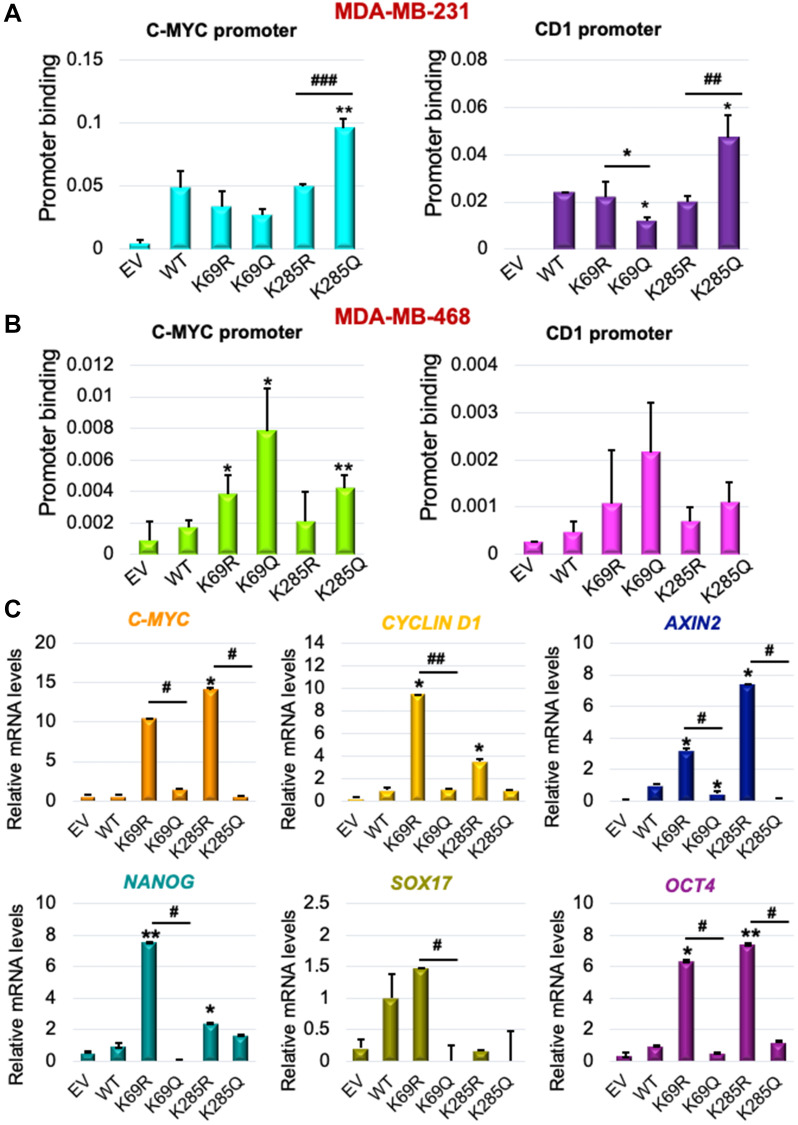
K69 and K285 lysine residues influences its binding and regulation of WNT target genes in triple-negative breast cancer models. Occupancy of HA-tagged DVL-1 at promoters of c-myc and cyclinD1 gene were analysed by real-time PCR. ChIP experiments for HA-tag were performed in (**A**) MDA-MB-231 and (**B**) MDA-MB-468 cells stably expressing EV (empty vector; negative control), HA-tagged wild-type DVL-1 (WT), HA-tagged deacetylation mutants (K69R and K285R), and HA-tagged acetylation mutants (K69Q and K285Q). The sign “*” represents significant change in HA-tagged DVL-1 binding between WT and mutants. (**C**) mRNA expression of WNT target genes such as *C-MYC, CYCLIND1, AXIN2*, *NANOG*, *SOX17,* and *OCT4*, was determined by qRT-PCR (mean ± SEM, normalized to β-actin as control) in triple-negative breast cancer cells (MDA-MB-231) stably expressing DVL-1 K69 and K285 mutants. The sign “^*^” represents significant change in gene expression between WT and mutants, while “^#^” represents significant change between R and Q mutants. All results are expressed as mean ± SEM and considered significant at ^*/#^ *p* < 0.05, ^**/##^ *p* < 0.01 and ^***/###^ *p* < 0.001.

Since K69 and K285 residues regulate DVL-1 nuclear localization [18], we next determined their impact on promoter binding to WNT target genes such as *c-myc* and *cyclinD1*. ChIP-quantitative real-time PCR (ChIP-qPCR) was performed for HA-tag in TNBC models. Cells were stably selected to express EV, WT-DVL-1, K>R and K>Q (point mutants at K69 or K285) in MDA-MB-231 and MDA-MB-468 cells. Dysregulation of Wnt target genes has been implicated in progression of TNBC [34, 35]. Interestingly, we found that relative to WT, multiple mutants showed decreased binding to both *c-myc* and *cyclinD1* promoter regions, while the K285Q showed increased binding relative to K285R at both promoters in MDA-MB-231 cells (Figure 4A, Supplementary Figure 13). Furthermore, similar experiments conducted in MDA-MB-468 cells showed that K69Q increased binding to both *c-myc* and *cyclinD1* relative to WT and K69R (Figure 4B). Our results demonstrate that both point mutants alter DVL-1 binding to Wnt target gene promoter regions with respect to WT.

To understand whether differential binding of WT and mutants correlated with a change in transcript levels of Wnt target genes, we isolated RNA from MDA-MB-231 cells stably expressing each of the indicated DVL-1 WT or point mutants (Supplementary Figure 14). Interestingly, we found that with enrichment in promoter binding in different TNBC cells, K69Q and K285Q corresponded with a statistically significant decrease in the *c-myc* and *CCND1* transcripts with respect to WT or K69R and K285R (Figure 4C). Furthermore, we observed that with K69Q and K285Q, there was a significant decrease in transcript levels of other Wnt target genes such as *AXIN2, NANOG, SOX17*, and *OCT4* in MDA-MB-231 cells with respect to K>R substitution. In contrast, K69R and K285R, mutants which were predominantly localized in the cytoplasm, corresponded with a significant increase in the levels of Wnt target genes transcripts. Collectively, these results from K285Q mutant shows the general trend that increased DVL-1 promoter occupancy correlates with decreased gene expression in MDA-MB-231 cells, suggesting that nuclear DVL-1 or K>Q mutants at K69, K285 residues may predominantly facilitate transcriptional repression of Wnt target genes frequently dysregulated in breast cancer.

### Conserved DVL-1 lysines influence migration, cell-cycle progression, proliferation and *in vivo* tumor growth in triple-negative breast cancer

**Figure 5:**
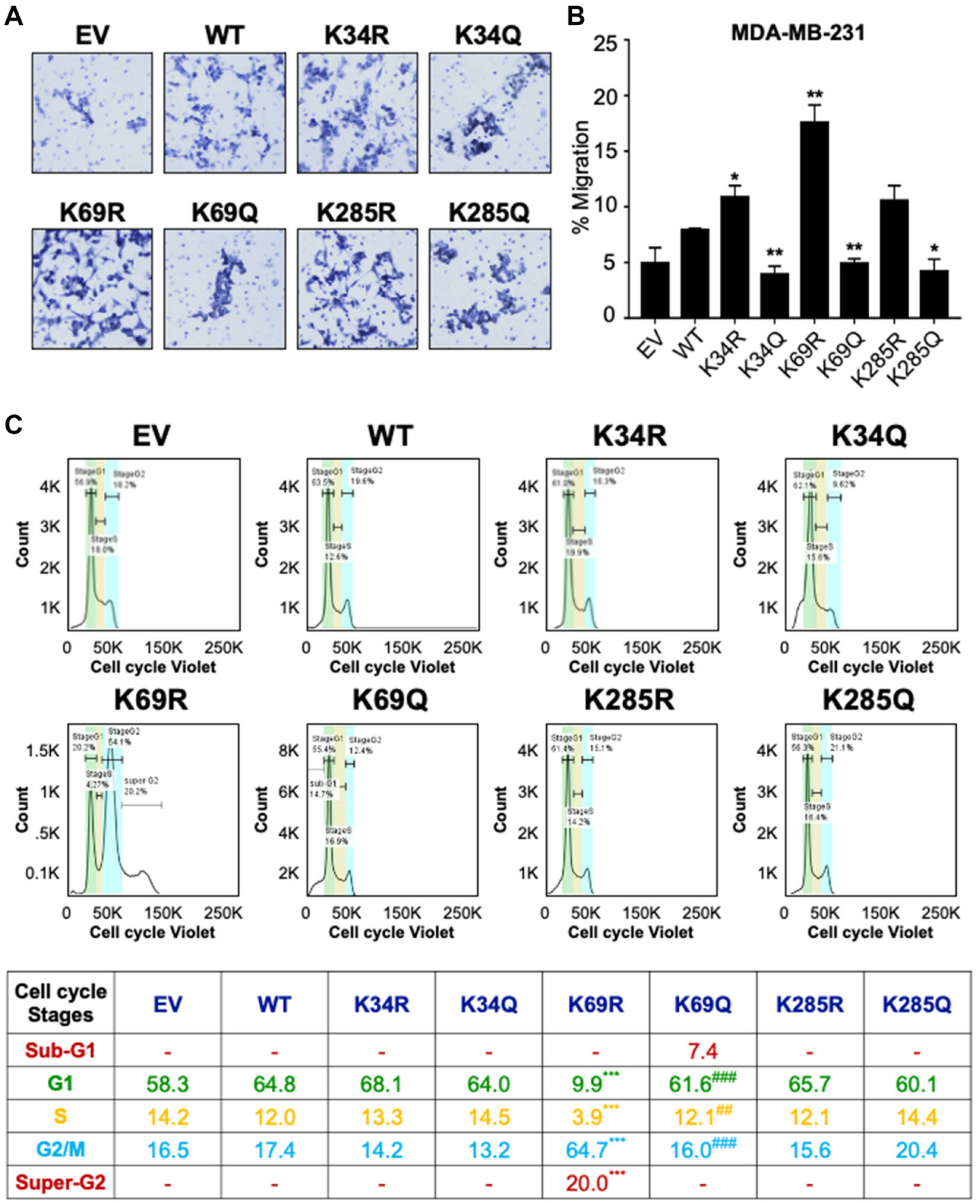
Conserved DVL-1 lysines at DIX and PDZ domain regulates cell migration and cell cycle progression in breast cancer cells. (**A**) MDA-MB-231 cells expressing different DVL-1 mutants were analyzed in a transwell migration assay and images were taken using Nikon microscope. (**B**) Changes in migration were measured after 20h and cells were counted using AdipoGauge software. All results are expressed as mean ± SD and considered significant at ^*^ *p* < 0.05 and ^**^ *p* < 0.01. (**C**) The effect of DVL-1 mutants was analyzed in a cell cycle assay using a Vybrant cell violet dye from Invitrogen followed by flow cytometry. The live cells are represented in the SSC versus FSC plots, which were further distributed among different cell cycle stages such as SubG1, G1, S, G2/M, and Super-G2 phase. Changes in cell cycle stages from four-independent experiments are represented in the table. The sign “^*^” represents significant change in gene expression with respect to WT-DVL1, while “^#^” represents significant change between R and Q mutants. All results are expressed as mean (*n* = 4) and considered significant at ^**/##^ *p* < 0.01 and ^***/###^ *p* < 0.001.

Wnt signaling is important for numerous cellular processes [1], and because DVL has been linked with migration in breast cancer cells [36, 37], we examined the influence of lysine point mutants on DVL-1 in regulating cell migration. Using transwell migration assays, MDA-MB-231 cells stably expressing EV, WT-DVL1, and point mutants, were allowed to migrate for 20 hours (Figure 5A). We observed that mutations on all three K-residues altered cell migration. In particular, the K69R increased migration relative to WT in MDA-MB-231 (Figure 5B). These results are the first to demonstrate that conserved DVL-1 lysines are important regulators of cell migration.

Additionally, previous reports have shown that DVL-3 regulates centrosomal cell-cycle stage by associating with linker proteins, suggesting that DVL is a crucial regulator of cell-cycle progression [38, 39]. In order to understand the effect of DVL-1 mutants on cell-cycle phases, we next examined the different stages in actively proliferating (live) MDA-MB-231 cells stably expressing WT and different DVL-1 mutants. Using Vybrant cell violet dye, the stained live cells were distributed into four cell-cycle population namely, Gap1 (G1), DNA synthesis (S), cell growth and mitosis (G2/M), G1-arrest (Sub-G1), and Super-G2/M population. DNA content analysis by flow cytometry from four-independent experiments revealed the presence of a sub-G1 aneuploid cell population in cells mimicking acetylation on the DIX domain, especially K69Q, suggesting that these cells could be more prone to apoptosis (Figure 5C, Supplementary Figure 15). By contrast, K69R cells showed a significantly higher G2/M and super-G2 population, a cell-cycle stage that allows DNA re-replication (polyploidy) compared to WT cells. Even though we did not notice any significant changes in cell population for other mutants compared to WT-DVL1 conditions, these results are consistent with the idea that conserved DIX domain lysine residues regulate cell-cycle progression. Here, we unravel a novel underlying mechanism that conserved lysine such as K69-DVL1 modulates cell-cycle transition in breast cancer model.

**Figure 6:**
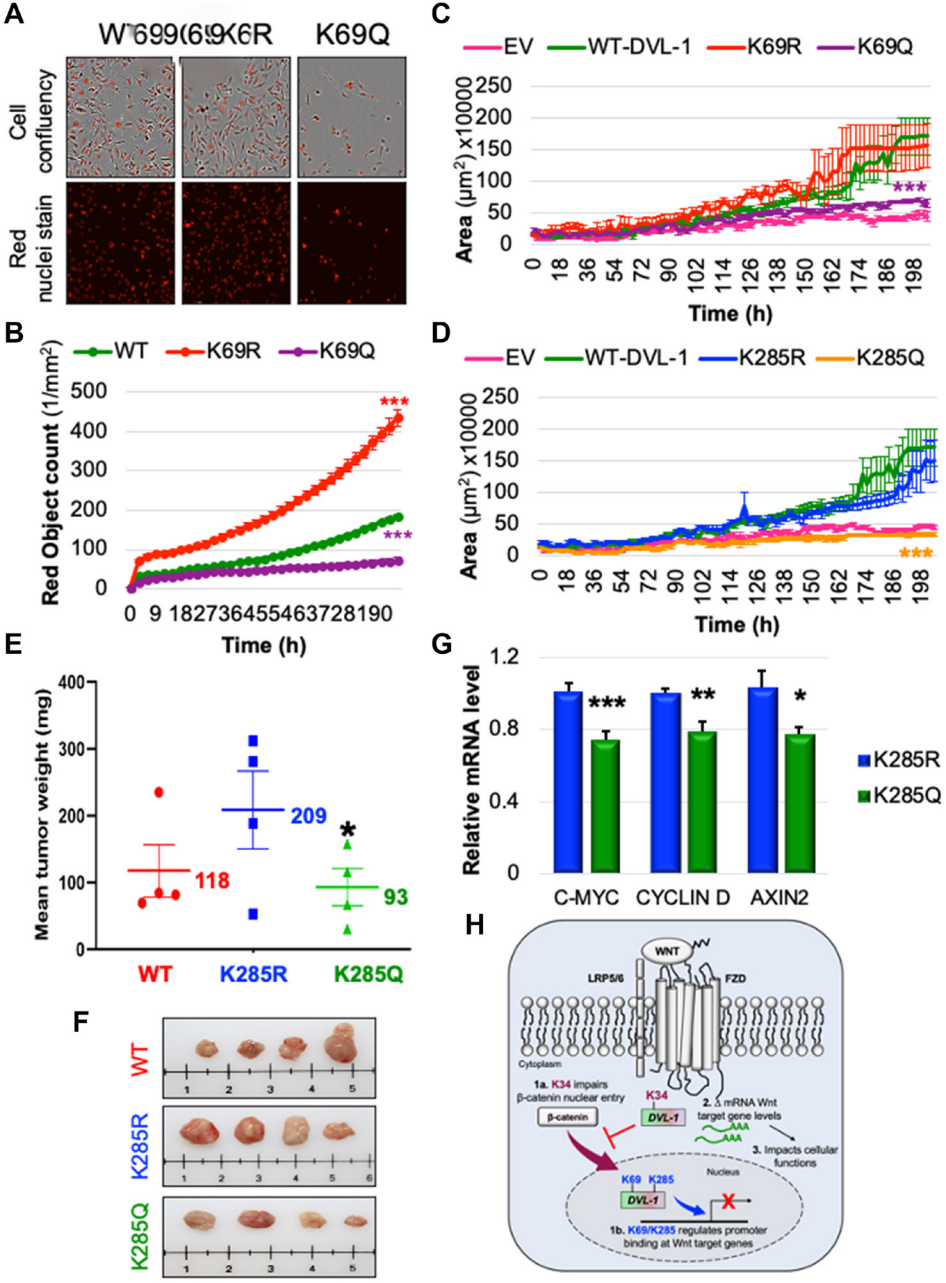
K69 and K285 residues significantly influence rate of cell proliferation and xenograft tumor growth respectively in MDA-MB-231 cells. (**A**) MDA MB-231 cells were seeded onto 96 well plates and grown in the presence of nuclear stain NucLight Rapid Red Reagent (Essen Biosciences). Cells were monitored and stained nuclei were quantified in real time by live cell imaging, with images captured every 2 hours for 120 hours. Images shown here represent confluency (upper panel) versus nuclei stain (bottom panel). (**B**) Each dot represents the nuclear counts at each time point. Data are shown as the mean nuclear counts (red fluorescent bodies/mm^2^) ± SEM and considered significant at ^***^ *p* < 0.001 compared to WT-DVL1. Spheroid formation assay was conducted in MDA-MB-231 cell lines stably expressing DVL-1 (**C**) K69 and (**D**) K285 mutants. Quantification of the area of spheroids is presented as the mean ± SD, where *n* ≥ 5 showing statistical difference after 200h ^***^ *p* < 0.001. (**E**) Mean tumor weight of WT-DVL1, K285R and K285Q MDA-MB-231 cells subcutaneously injected in mammary fat pad of immunocompromised mice (*n* = 4 per group, “^*^” represents significant change between R and Q mutants where ^*^ *p* < 0.05). (**F**) Representative images for tumors from mice receiving WT-DVL1, K285R and K285Q MDA-MB-231 cells (top to bottom). (**G**) mRNA expression of WNT target genes such as *CMYC*, *CYCLIND1*, and *AXIN2* was determined by qRT-PCR (mean ± SEM, normalized to β-actin as control) using tumor obtained from xenograft studies, where *n* = 4. The sign “^*^” represents significant change in gene expression. All results are expressed as mean ± SEM and considered significant at ^*^ *p* < 0.05, ^**^ *p* < 0.01 and ^***^ *p* < 0.001. (**H**) Schematic representation of the functional significance of lysine residues on the DIX and PDZ domains od DVL-1 with respect of Wnt signaling. While highly conserved K34 residue is critical for the entry of β-catenin into nucleus, K69/K285 residue regulate DVL-1 binding to promoter binding at Wnt target genes, and repressing their expression thereby impact broad cellular functions.

Since we were observing striking phenotypic differences with the PTM mimics at the K69 site, we performed another cell-biology based assay to measure the rate of cell proliferation by staining the nuclei of WT, K69R, and K69Q cells by live cell imaging. Consistent with other assays, we found that the K69R cells showed a significant increase in proliferation compared to WT cells (which supports the flow cytometry data, where K69R has significantly high G2/M and super-G2 population), while the K69Q cells resulted in a significant reduction in cell proliferation compared to WT and K69R. Together, this suggests that post-translational regulation of specific DVL-1 lysines control cellular functions and oncogenic signaling (Figure 6A–6B). In addition, we performed 3D-spheroid assay to understand the role of DVL-1 and key lysines in regulating this cancer hallmark. Interestingly, we observed that in MDA-MB-231 cells, both K69Q/K285Q significantly reduced 3D-spheroid growth over the course of 200 hours *in vitro* (Figure 6C–6D, Supplementary Figure 13C).

Furthermore, we extended our analysis to assess the impact of DVL-1 and K285 on tumor growth *in vivo*. We chose to perform *in vivo* experiments with a focus on K285 for three reasons: First, acetylation of K285 was mapped only in cancer cells, hence making Lys-285 acetylation unique within the cancer-context [18]; second, the K285 residue is located on the highly conserved PDZ domain of DVL proteins, a domain considered as a good therapeutic target [40–42]; and lastly, based on *in vitro* experiments, K285 mutants had the most robust effect on 3D spheroid formation compared to WT conditions. Therefore, we performed xenograft tumor growth experiment by injecting MDA-MB-231 stably expressing a WT-DVL1, K285R vs. K285Q in the mammary fat pad of athymic nude mice. Interestingly, we observed that mice receiving K285R cells had increased *in vivo* tumor growth compared to WT-DVL1 in immunocompromised mice. Moreover, there was a significant decrease in the final tumor weight in K285Q compared to K285R (Figure 6E–6F). Lastly, to validate the *in vitro* findings on Wnt-regulated genes, we performed RNA expression analysis from excised xenograft tumors. We observed similar trends, where K285R tumors significantly increased the levels of Wnt gene transcripts including *c-myc, CCND1*, and *Axin2* with respect to K285Q tumors (Figure 6G). Collectively, these results demonstrate that DVL1-K285Q significantly reduces tumor growth *in vivo*, possibly by altering Wnt target gene expression in TNBC model.

Taken together, these findings indicate that the conserved lysines on DVL-1 proteins are critical regulators of Wnt signaling as well as physiologically relevant, wherein it regulates oncogenic biological functions such as 3D-spheroid formation, transwell migration, cell-cycle phases, proliferation, and xenograft tumor growth in triple-negative breast cancer (Figure 6H).

## DISCUSSION

Dishevelled proteins are integral to Wnt signaling and regulate cell-to-cell communication important for different cellular processes such as cell growth and differentiation. Depending on the nature of the upstream signal, DVL can engage canonical or non-canonical branches of the Wnt pathway. One question that continues to linger in the field is what regulates DVL-1-mediated engagement of canonical and non-canonical Wnt signaling? Here, we report for the first time that conserved lysines which undergo post-translational acetylation control critical aspects of DVL-1 function. Hence, one may reason that post-translational acetylation or any other PTM which changes the charge at any of the three key lysines could play a role in determining which downstream pathway is activated. The charge of these three lysine residues likely plays an important role given the fact that substitution with both R and Q show very different phenotypes, suggesting that the phenotypic change is not simply due to the absence of lysine at a specific position. Increasing evidence suggests that several Wnt pathway components undergo post-translational modifications that are essential for fine tuning different cellular functions [43, 44]. Highly conserved structural domains of DVL proteins are subjected to PTMs such as phosphorylation, methylation, and ubiquitination [45]. Various elegant studies have demonstrated that kinases deposit site-specific phosphorylation at serine, tyrosine, and threonine residues which primes Wnt pathway activation and elicits diverse biological responses [46, 47]. Also, methylation of arginine R271 on DVL-3 has been reported to inhibit Wnt pathway by reduced membrane localization [17, 48]. Furthermore, DVL is known to interact with proteins such as NEDDL4, HUWE1, ITCH, KHLH12-cullin3, Smurf2, and WWP2, which ubiquitinate and target K-residues on DVL for proteasomal degradation, causing an inhibition of downstream Wnt signaling functions such as axis duplication in xenopus embryo and affecting autophagy-lysosome pathway [13–15, 20]. On the other hand, ubiquitination at multiple lysines in the DIX domain can be reversed by various deubiquitinases (DUBs), including a tumor suppressor CYLD, allowing formation of signalosome via DVL polymerization [49].

While much more work has been done with respect to post-translational regulation of DVL-2 and DVL-3 as described above, significantly less attention has been given to DVL-1. Herein, we focus on elucidating the role of three key DVL-1 lysines, present within the DIX and PDZ domains, which we recently demonstrated were regulated via post-translational lysine acetylation. Our findings clearly demonstrate that K>Q substitutions on highly conserved DVL-1 lysine residues (K34, K69, K285) modulate Wnt signaling via different mechanisms impacting broad cellular functions. Using *in vitro* and PDX models we demonstrated that DVL undergoes acetylation at 12 lysine residues, present in conserved DIX, PDZ and DEP domains. Previously we had demonstrated that K69 and K285 residues control nuclear localization of DVL-1 in TNBC models. These acetylation sites were identified on endogenous DVL proteins in TNBC models treated with vehicle control or FDA-approved or pre-clinical lysine deacetylase inhibitors (KDACi). Interestingly, we noted that DVL-1 K34 acetylation occurred across multiple contexts including in cell lines, in patient-derived xenograft tumors, and at different oxygen tensions and treatment conditions. Here we observed that, unlike K69 and K285, the K34 residue does not regulate cytosolic/nuclear DVL-1 ratios in breast cancer or HEK293 cells. Instead, we noted that the K34 residue is important for regulating DVL-1 affinity to β-catenin and the FZD7 receptor. Moreover, our immunofluorescence experiments suggested that K34 plays a role regulating β-catenin levels in the cytoplasm relative to other cellular compartments such as within the nucleus or on promoters of key Wnt target genes that are important for cancer growth. Hence, these results uncover novel molecular mechanism(s) that could contribute to Wnt pathway regulation by modulating β-catenin cellular distribution. Interestingly, K34Q downregulates both Wnt target gene expression as well as cell migration, which might suggest that K34 may be playing a role in mediating Wnt signals into some other branch such as the Wnt/Ca2+ pathway. However, at this stage we cannot fully explain this gap in knowledge. Given that these three lysines (K34 and K285 in particular, see Table 1) might be subject to other PTMs including methylation, ubiquitination, and sumoylation, the effects we observe in our assays might not only be due to altered acetylation, but also due to other PTMs such as ubiquitination [16].

In addition, further analysis of K69 and K285 acetylation revealed that not only do these residues act as a regulatory switch for DVL-1 nuclear translocation, but they also impact DVL-1 binding to different gene promoters and regulate Wnt target gene expression in TNBC models. While this study focuses on selective Wnt target genes, the role of nuclear DVL seems to be considerably more expansive than previously considered. Our group has recently published a comprehensive DVL ChIP Seq study to understand the genome-wide binding patterns of DVL-3 in human breast cancer models [24]. In this study, we demonstrated that DVL-3 binds to a histone methyltransferase KMT2D resulting in alteration of H3K4me3 marks at DVL3-target regulatory regions, hence regulating expression of genes which are critical for immune response, signal transduction and metabolism. Herein, we share a snapshot of DVL-1 mediated regulation of Wnt target genes, where we notice a general trend that increased DVL-1 promoter occupancy correlates with decreased gene expression, suggesting that nuclear DVL-1 is frequently linked with transcriptional repression of Wnt target genes. Interestingly, our observations regarding nuclear DVL-1 are similar to one of the previously published studies where nuclear DVL-1 exerts tumor suppressive effects by inhibiting transcription of ribosomal DNA in breast cancer model [26]. Interestingly, K>Q substitution of conserved K69 and K285 residues also significantly reduce Wnt target gene transcript levels, in TNBC cell lines and xenograft tumors.

Early discoveries associated the phenotype of *DVL* with disorientation in *Drosophila* wing hair and segment polarity in *Xenopus* embryo, and social abnormalities in mice [50, 51]. In humans, frameshift mutation on C-terminus of *DVL* is associated with Robinow Syndrome, a disorder characterized by skeletal abnormalities [52, 53]. Interestingly, our functional assays conducted in cancer models demonstrate that key lysine residues subject to acetylation play a critical role in modulating cell migration, proliferation, cell cycle progression, and *in vivo* tumor growth, further bolstering the concept that these novel DVL-1 PTMs are functionally significant and could impact Wnt signaling branches and multiple cellular processes linked with tumorigenesis.

## MATERIALS AND METHODS

### Cell culture

Cell lines used in this manuscript (MDA-MB-231, MDA-MB-468, and HEK293) were purchased from ATCC. The cells were authenticated utilizing STR technology and used in a low passage (<20) within 6 months after receipt or resuscitation. MDA-MB-231 and HEK293 were grown in DMEM (Gibco), while MDA-MB-468 were grown in RPMI 1640 (Gibco), supplemented with 10% fetal bovine serum and 1% penicillin/streptomycin (Invitrogen). HEK293T-Rex DVL TKO cells were a kind gift from Vítězslav Bryja lab.

### Immunofluorescence staining in primary human breast cancer tissues

Paraffinized tissue blocks from different subtypes of breast cancer patients were obtained from a de-identified tumor repository at TTUHSC Pathology department. The tissues were used to identify DVL-1 expression by immunofluorescence staining. The slides were blocked with 1% phosphate buffer saline (PBS) (Thermo Fisher Scientific) at room temperature, followed by incubation with 0.1% Saponin (Sigma), and 5% bovine serum albumin (BSA) (Sigma) for 1 hour and then incubated overnight at 4°C with DVL-1 rabbit polyclonal antibody (D3570; Sigma) at 1:250 dilution dissolved in blocking buffer (1X PBS, 0.1% Saponin, 5% BSA). The following day, slides were incubated with an Alexa fluorophore-conjugated secondary Cy3TM donkey anti-rabbit IgG (Thermo Fisher Scientific) at 1:300 dilution in blocking buffer against the rabbit primary antibodies for 2 hours at room temperature and mounted with DAPI (Life Technologies) overnight in the dark. The slides were sealed and stored at 4^°^C and imaged using a laser scanning confocal microscope Nikon T-1E at 60X resolution. The images were then analyzed using AdipoGauge software for Windows (Version 2.0) [54]. For statistical analysis, multiple Welch *t*-test was performed to compare any significant differences between DVL-1 expression in different breast cancer subtypes (*n* ≥ 3; ^*^ *p* < 0.05; ^#^ *p* < 0.1).

### DVL-1 stable knock-down

TNBC cells were transduced with pLKO.1-puro based shRNA MISSION lentiviral particles for DVL-1 (TRCN0000441114, Sigma), and non-target shRNA control (SHC002V, Sigma). After 24 hours of plating, cells were transduced with hexadimethrine bromide (Sigma, H9268) at a final concentration of 8 μg/ml, followed by addition of appropriate amounts of viral particles to the media. After 24 hours the media was replaced and 1 μg/ml of puromycin (Gibco, A11138-03) was added for selection. Puromycin-containing media was replaced every 3–4 days until total selection was achieved.

### DVL-1 stable over-expression

350,000 cells were plated in a p6-well dish and transfected with 1 μg DNA using Lipofectamine3000 reagent (Thermo Scientific). Next day, media was replenished and selected using 1 mg/ml G418 antibiotic until no cells were remaining in non-transfected control. Plasmid transfections were performed using Lipofectamine 3000 reagent (Thermo Scientific) according to manufacturer’s protocol. For the experimental analysis, one clone for each variant was used in multiple cell lines. The clones were chosen based on the relative levels of HA-tag and DVL-1 levels.

### Immunoblots

Antibodies used for immunoblots are: DVL-1 (D3570; Sigma), HA (cst-3724; Cell Signaling), active/non-phosphorylated β-catenin (cst-8814; Cell Signaling), total β-catenin (sc-7963; Santa Cruz), DVL2 (sc-271319; Santa Cruz), DVL-3 (SAB4200007; Sigma), CK1Ɛ (sc-373912; Santa Cruz), Lamin A/C (cst-4777; Cell Signaling), β-tubulin (T7816; Sigma), and GAPDH (sc-47724; Santa Cruz), and β-actin (sc-47778; Santa Cruz). Membranes were incubated with blocking buffer (5% milk/TBST) with primary antibody overnight at 4°C. This was followed by probing the membranes with HRP-conjugated secondary antibodies for 1 hour/room temperature (RT). Lastly, the signals were visualization using ECL reagent (Thermo Scientific) and imaged in Azure C300 gel imaging system (Azure Biosystems).

### *In silico* analysis

DVL-1 protein sequence was acquired from NCBI browser. Multiple sequence alignment of DVL-1, DVL-2, DVL-3 across different organisms was performed using Clustal Omega software (https://www.ebi.ac.uk/Tools/msa/clustalo/). The structure and acetylated lysine residues on DVL-1 DIX domain were modeled using the PYMOL software for Supplementary Figures. ModPred tool was used to predict and analyze concurrently several PTM modifications on DVL-1 protein and specific lysine residues on the DIX and PDZ domain as shown in Table 1 [55].

### Fluorescence microscopy

The protocol for fluorescence microscopy was followed as previously published [18]. Additionally, the primary antibodies used were: HA (cst-3724; Cell Signaling), β-catenin (sc-7963; Santa Cruz); while the secondary antibodies: Alexa flour 568 #A11036, and phalloidin 488 #A12379 (Thermo Scientific).

### Co-immunoprecipitation

Co-IP lysis buffer (25mM Tris, pH 7.4, 150 mM NaCl, 1 mM EDTA, 1% NP-40, 5% glycerol and Protease inhibitor cocktail) was used for cell lysis. 2 mg of protein was incubated with 2 μg of DVL-1 antibody (D3570; Sigma), or HA-tag antibody (cst-3724; Cell Signaling), Rabbit-IgG for 2 hours at 4°C. This was followed by incubation with Protein A Dynabeads (Life Technologies) for 2 hours at 4°C. The antibody-antigen complex was washed with lysis buffer, four times with gentle agitation. For elution of complex from beads, 4x sample buffer (Invitrogen) was used followed by Western blotting along with WCE.

### Subcellular fractionation

Cells used for nuclear and cytoplasmic fractionation were HEK293 stably expressing EV, WT, K34R, and K34Q mutants. Nuclear and cytosolic extracts were prepared using NE-PER kit (Thermo Scientific).

### Liquid chromatography/mass spectrometry (LC-MS/MS)

Patient-derived TNBC tumors were obtained from TTUHSC Cancer Center and harvested in RIPA buffer (with complete protease inhibitor cocktail, 1 μM TrichostatinA and 1mM nicotinamide), followed by homogenisation using the M24 tissue homogeniser (10 cycles for 30 sec homogenization followed by 5 mins rest on ice). Protein concentration was quantified by the BCA method and incubated with 10 μg of anti-DVL-1 antibody (D3570; Sigma) overnight at 4°C. Protein A dynabeads (Invitrogen) were added to the immune-complex and incubated for 2 hours/4°C. Beads were washed four times with RIPA lysis buffer followed by 2 washes with autoclaved water, with gentle agitation for 5 mins per wash. Dry beads were shipped to Applied Biomics Inc. (Hayward, CA) for acetylation site identification by LC-MS/MS mass spectrometry. For more details about the protocol please see Sharma et al. 2019 [18].

### mRNA expression analysis

For RNA extraction from xenograft tumors, 20 mg of tumor tissue was homogenized with 2.8 mm ceramic beads (13114-325, Qiagen Cat) using FastPrep-24 (6004-500, MPbio). Total RNA was isolated from cells or tumors using a Bio-Rad RNA extraction kit. SuperScriptIII Reverse Transcriptase (ThermoFisher) was used to reverse-transcribe 2 μg of total RNA to synthesize first-strand of complementary DNA (cDNA). Intron-spanning primers were designed for each specific target DNA and gene expression measured by either endpoint-PCR or real-time qRT-PCR.

### End-point PCR and quantitative real-time qRT-PCR

The PCR protocol was followed as previously published by our group [18]. The RNA primers are listed in Table 2A.

**Table 2:** List of RNA primers for Wnt gene expression and ChIP-primers used in the study

Table 2A: List of RNA primers		
Gene	Forward primer	Reverse primer
*AXIN1*	ATGGAGCTCTCCGAGACAGA	ACCAGCCTATCAGTCCACCT
*AXIN2*	TATCCAGTGATGCGCTGACG	CGGTGGGTTCTCGGGAAATG
*SOX17*	GTGGACCGCACGGAATTTG	GGAGATTCACACCGGAGTCA
*NANOG*	TTTGTGGGCCTGAAGAAAACT	AGGGCTGTCCTGAATAAGCAG
*POU5F1(OCT4)*	CTTGAATCCCGAATGGAAAGGG	GTGTATATCCCAGGGTGATCCTC
*FZD7*	CGCGGCCGCTCCGCTTTC	GCGCTCGCACAGAGAACGACA
*CD1*	TCTTGTGCCACAGATGTGAAGTT	GAGGCAGTCTGGGTCACACTT
*CMYC*	AAACACAAACTTGAACAGCTAC	ATTTGAGGCAGTTTACATTATGG
*BACTIN*	GGACTTCGAGCAAGAGATGG	AGCACTGTGTTGGCGTACAG
*B2M*	CACCCCCACTGAAAAAGATG	ATATTAAAAAGCAAGCAGCAGAA
**Table 2B: List of ChIP primers**		
**Gene**	**Forward primer**	**Reverse primer**
*CD1*	CGCTCCCATTCTCTGCCGGG	CCGCGCTCCCTCGCGCTCTT
*CMYC*	CGTTTTCCTCCTTATGCCTCTATC	GTACCAGGCTGCAGGGCGCCTCGCT

### Chromatin immunoprecipitation

For more details about the ChIP protocol please see Castro-Piedras et al. (2018) [25]. The ChIP primers are listed in Table 2B. For more details, see Supplementary Figure 16.

### Spheroid proliferation assay

Breast cancer cells were seeded (100 μL per well) at a density of 2 × 10^4^ per well into a 96-well ULA plate, forming spheroids with diameter 200–500 μm after three days. MDA-MD-231 required the addition of 1% Matrigel (35234; Corning) to promote spheroid formation. The ULA plate was centrifuged (1000 RPM, 10 minutes) at room temperature and then placed into the IncuCyte™ ZOOM Live-Cell Analysis System (Essen Bioscience) to monitor the process of spheroid formation and growth. The plate was allowed to equilibrate for 20 minutes, and the scan was scheduled as 24-hour repeat scanning (1 image per well every 4 hours for 200 hours) in the IncuCyte™ software using the objective 10x.

### Transwell migration assay

MDA-MB-231 cells were trypsinized and re-suspended in serum-free DMEM media, and from this suspension 500 μl (2.5 × 10^4^ cells) were placed on the upper chamber on uncoated 24-well 8 μm inserts (BD Falcon). The bottom chamber was supplemented with 750 μl of complete DMEM media (supplemented with serum) as chemoattractant. Cells were allowed to migrate for 20 hours at 37°C, 5% CO_2_ atmosphere. Following incubation, media from upper chamber was carefully removed without touching the bottom surface of the insert using a pipette. Non-migrant cells were gently swabbed off the top insert and discarded. The inserts were washed with 1X PBS and cells were fixed in 70% ethanol for 10 mins at RT. Cells were then stained with 1% crystal violet for 45 mins. The inserts were allowed to dry at RT and representative images for each cell type were taken in triplicates, and imaged on an (10x) Nikon light inverted microscope. Collected images were then analyzed for counting cells using AdipoGauge software for Windows (Version 2.0) [54]. For statistical analysis, multiple Welch *t*-test was performed to compare any significant differences between WT and DVL-1 mutants (*n* = 3; ^*^ *p* < 0.05; ^#^ *p* < 0.1).

### Cell cycle analysis

For each cell type, 1 × 10^6^ cells/mL were stained according to the manufacturer’s protocol with Vybrant DyeCycle™ Violet Stain (Invitrogen) at a concentration of 5 uM and data was collected on a BDFortessa. Collected data was then analyzed using FlowJo v7.6.5 (Becton Dickinson).

### Cell proliferation studies using IncuCyte

Triple-negative breast cancer cells stably expressing DVL-1 knockdown, wild-type DVL-1 or point mutants in triple-negative breast cancer cells were maintained in DMEM, 10% FBS, 1% P/S and appropriate selective agent in T-75 flasks. Cells were seeded onto 96 well plates and grown in the presence of nuclear stain NucLight Rapid Red Reagent (Essen Biosciences) at 1:1000 dilution. Cells were monitored and cell confluence or stained nuclei were quantified in real time by live cell imaging at 37°C, 5% CO_2_, using the IncuCyte ZOOM (Essen Biosciences). Images were collected every 2 hours for 120 hours.

### Nude mice xenograft model

All the athymic NU/J mice (002019, Jackson Laboratory) were raised in a pathogen-free environment within the animal laboratory under a 12-hour light/12-hour dark cycle. Food and water were provided ad libitum. 6 million (resuspended in 200 μL 1X PBS) MDA-MB-231 cells stably expressing empty vector (EV), wild-type DVL-1 (WT), K285R and K285Q were subcutaneously injected into the fourth mammary fat pad on the female mice right side. The tumor size of each mouse was monitored using digital caliper once a week and tumor volume was calculated using the formulae (L × W × W)/2. The weight of the tumor excised from each of the mice was measured after euthanasia. All animal care and experimental procedures used were approved and conducted in accordance with the National Institutes of Health accepted guidelines [56] and with approval from the Institutional Animal Care and Use Committee at Texas Tech University Health Sciences Center.

### Quantification and statistical analysis

Statistical analysis was performed between two biological groups using Welch *t*-test and expressed as mean ± SEM. All results are considered significant at ^*/#^ *p* < 0.05, ^**/##^ *p* < 0.01 and ^***/###^ *p* < 0.001. Each figure with statistical analysis represents *n* ≥ 3, where n represents biological replicates. Details of statistical analysis can also be found in figure legends.

## CONCLUSIONS

In conclusion, this study provides new insights into the potential role of three highly conserved K-residues of DVL-1 in oncogenic signaling and broad cellular functions. First, using pateint tissue and xenograft *in-vivo* studies we highlight the oncogenic role of DVL-1 in TNBC models. Furthermore, we uncover the role of three lysines on structural domains of DVL-1 and characterize their impact on several fundamental cellular processes such as transcription of stem-cell and cancer-associated genes, 3D-spheroid growth, cell migration, and cell cycle progression. Thus, the modifications on domain-specific lysines could be subject to regulation by therapeutics already in clinical use or they could serve as potential therapeutic targets to modulate dysfunctional Wnt signaling.

## SUPPLEMENTARY MATERIALS


