# Supplementary material for: Dishevelled-1 DIX and PDZ domain lysine residues regulate oncogenic Wnt signaling
Source: Oncotarget. 2021 Oct 26;12(22):2234–51. doi: 10.18632/oncotarget.28089 (PMC8555683; doi:10.18632/oncotarget.28089)
Supplement: Supplementary file 1 [file oncotarget-12-2234-s001.pdf]

Dishevelled-1 DIX and PDZ domain lysine residues regulate oncogenic Wnt signaling

SUPPLEMENTARY MATERIALS

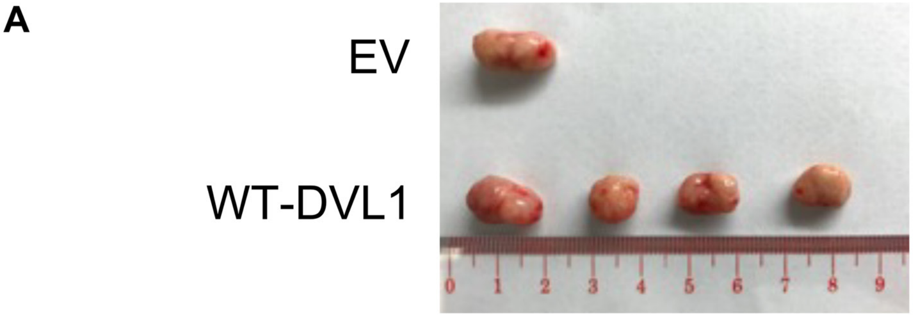

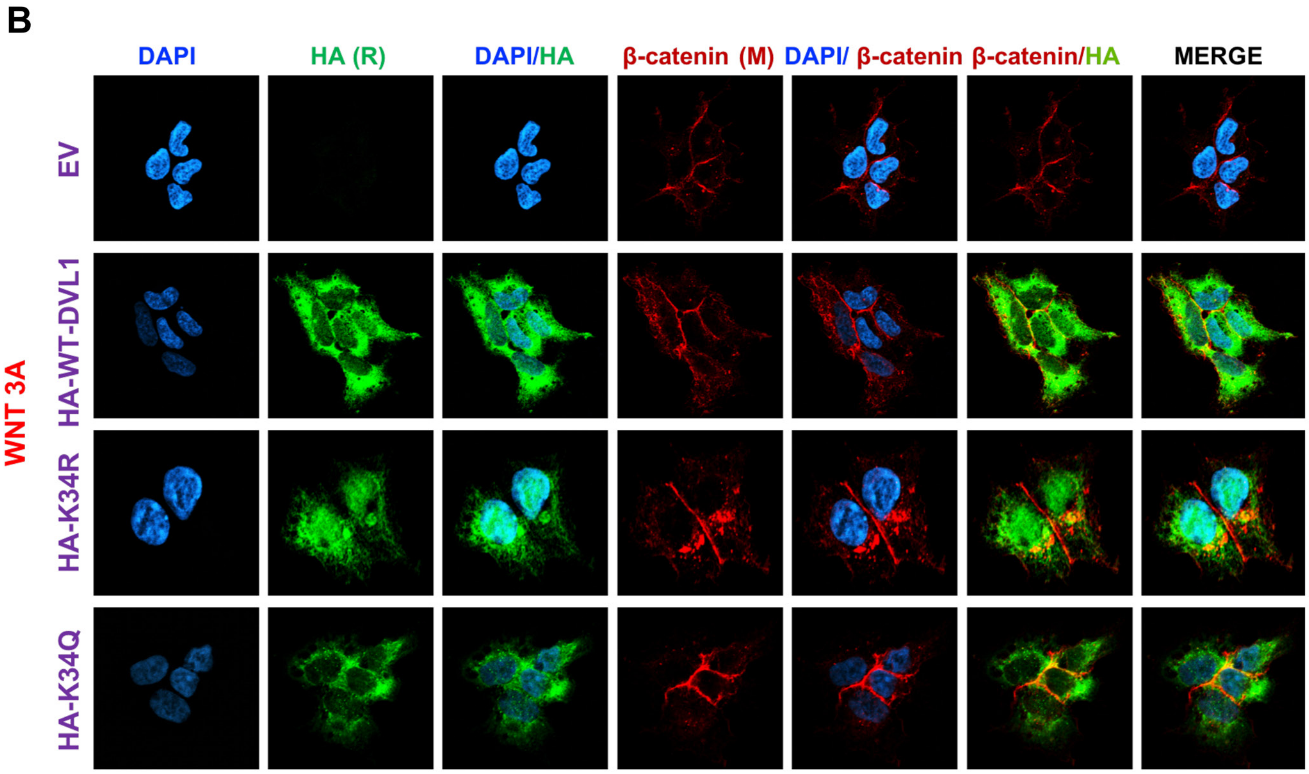

Supplementary Figure 1: (**A**) Representative images for tumors from mice receiving EV and WT-DVL1 cells. (**B**) Effect of Wnt3a treatment on β-catenin localization in WT and K34 mutants. Immunofluorescence staining was conducted as indicated to analyse relative localization of HA-tagged DVL1 proteins and β-catenin. Merge of nuclear staining (DAPI, blue), N-HA-DVL-1 (green), β-catenin (red) proteins is shown as DAPI/HA and DAPI/β-catenin for each of the mutant. Merge of β-catenin (red) and N-HA-DVL-1 (green) proteins is shown as β-catenin/HA for each of the mutant.

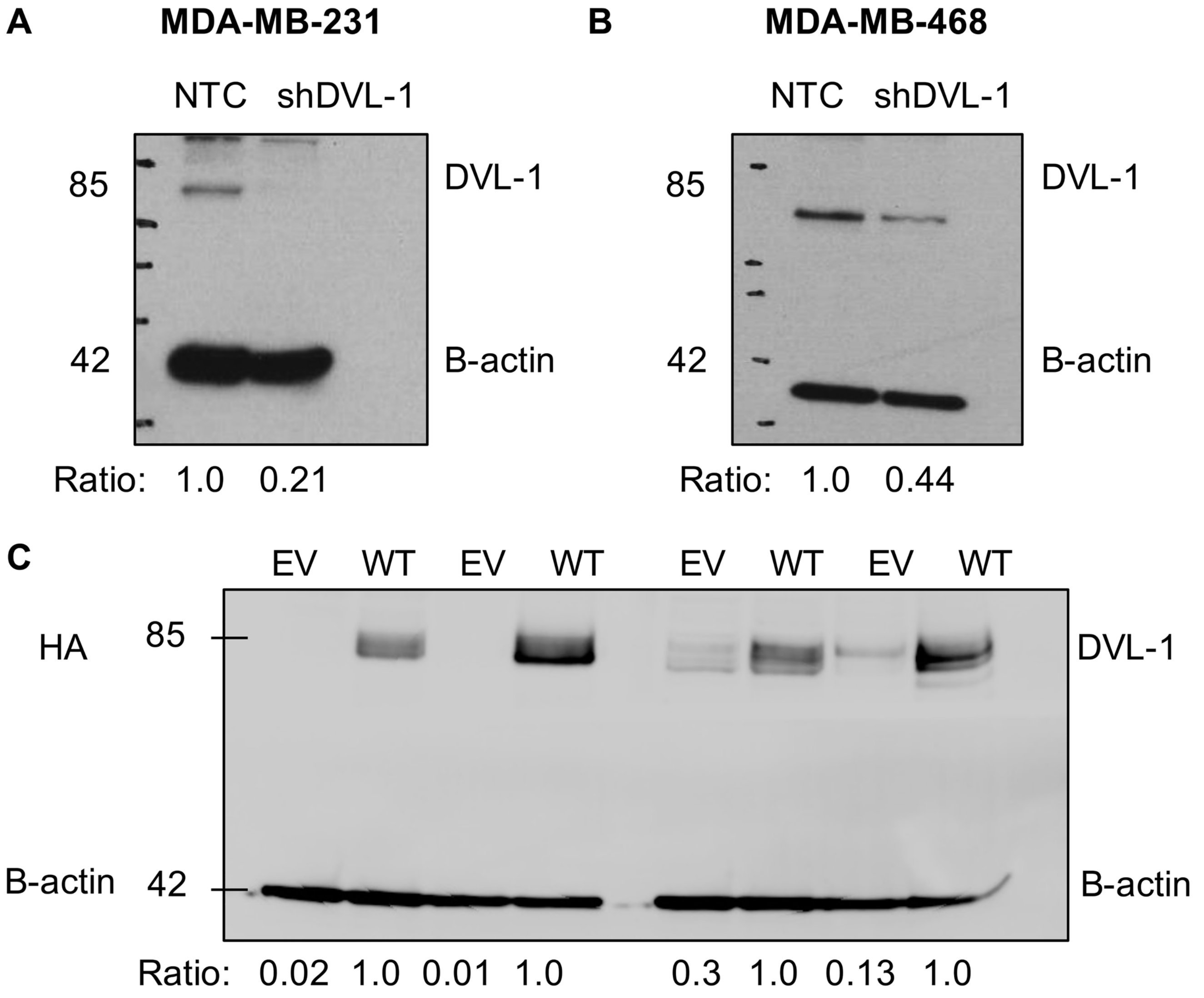

Supplementary Figure 2: Original western blots for Figure 1. Uncropped western blot images for DVL-1 protein expression in NTC and shDVL1 knockdown in (**A**) MDA-MB-231 and (**B**) MDA-MB-468 cells. (**C**) Original western blot for DVL-1 expression in empty vector (EV) versus wild-type DVL1 gain-of-function in MDA-MB-231.

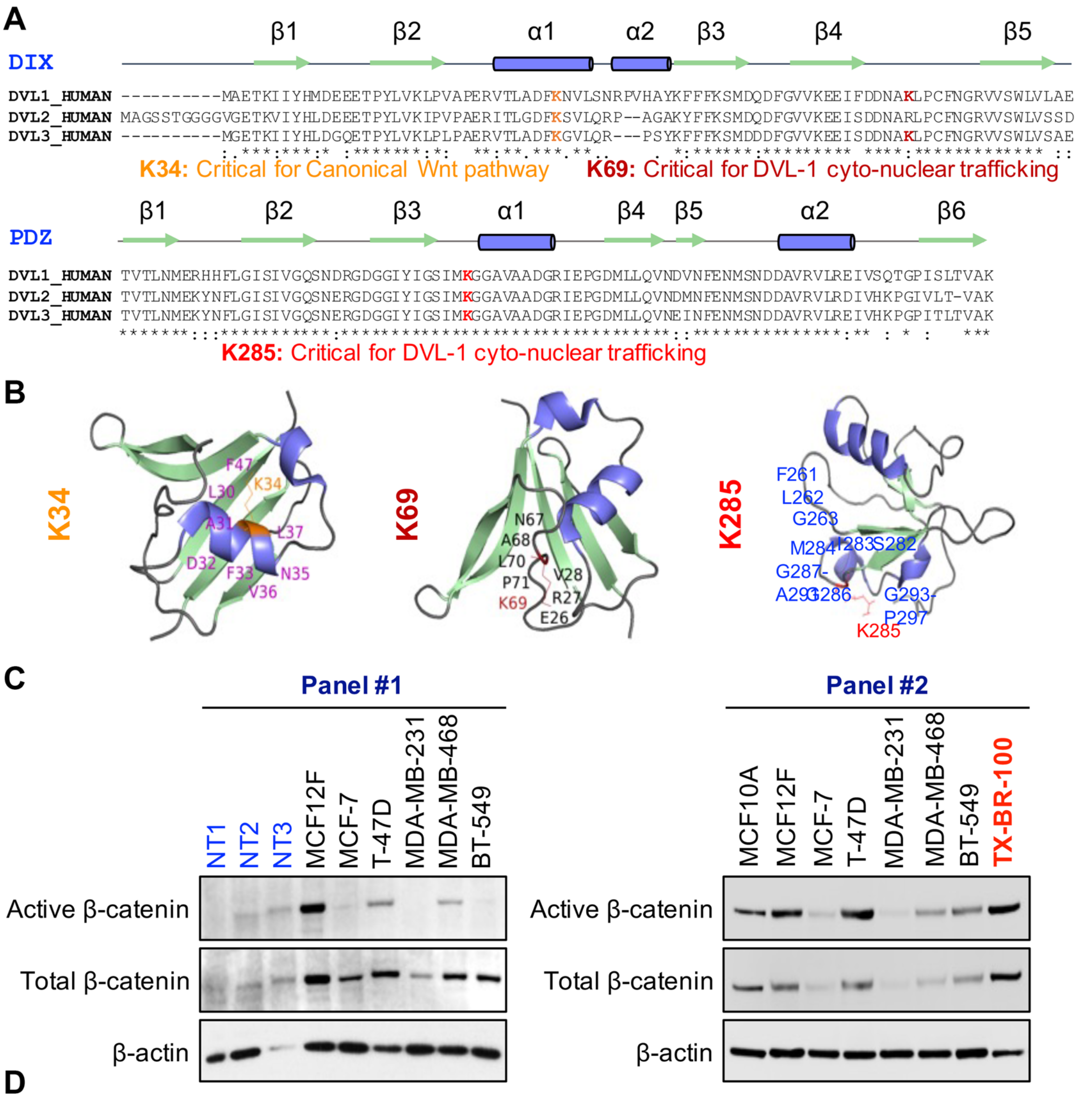

D

Sample Name	Acetylation-Peptide Sequence	K-Ac
TNBC-PDX-Exp#1	VTLADFK	K34
	KYASSLLK	K469, K476
	IIYHMDEEETPYLVK	K20
	QSFQKAMGNPCEFFVDI	K682
TNBC-PDX-Exp#2	VTLADFK	K34
	QSFQKAMGNPCEFFVDI	K682

Supplementary Figure 3: Highly conserved lysine residues undergo acetylation on DVL-1 proteins. (**A**) Schematic representation of the DIX and PDZ domains of DVL proteins, followed by sequence alignment of the three human DVL isoforms. The conserved acetylated lysine residues are highlighted in as K34 (orange), K69 (dark red), K285 (bright red). The green arrow represents a-helices and the purple loop represents b-sheets. (**B**) Crystal structure of DVL-1 DIX (PDB: 3PZ8) and PDZ domain (PDB: 1MC7). The lysine residues – K34, K69, and K285 are highlighted along with other amino acid residues in 4A proximity. (**C**) Expression of Wnt pathway components in breast cancer cell lines. Total protein was extracted from two panels of different breast cell lines: human normal tissue (NT1, NT2, NT3), non-cancer mammary epithelial cell line (MCF10A and MCF12F), hormone receptor positive breast cancer cells (MCF-7, T-47D), triple-negative breast cancer cell lines (MDA-MB-231, MDA-MB-468 and BT-549), and patient-derived xenograft cell line (TX-BR-100). Protein expression patterns of endogenous active β- catenin, total β-catenin and β-actin were analysed by western blotting in breast cells lines as described above. (**D**) Putative lysine residues acetylated on endogenous DVL-1 in triple-negative breast cancer patient-derived xenograft tumors (TNBC PDX-tumors) along with their representative peptide as detected by two-independent liquid chromatography mass spectrometry (LC-MS/MS) analyses.

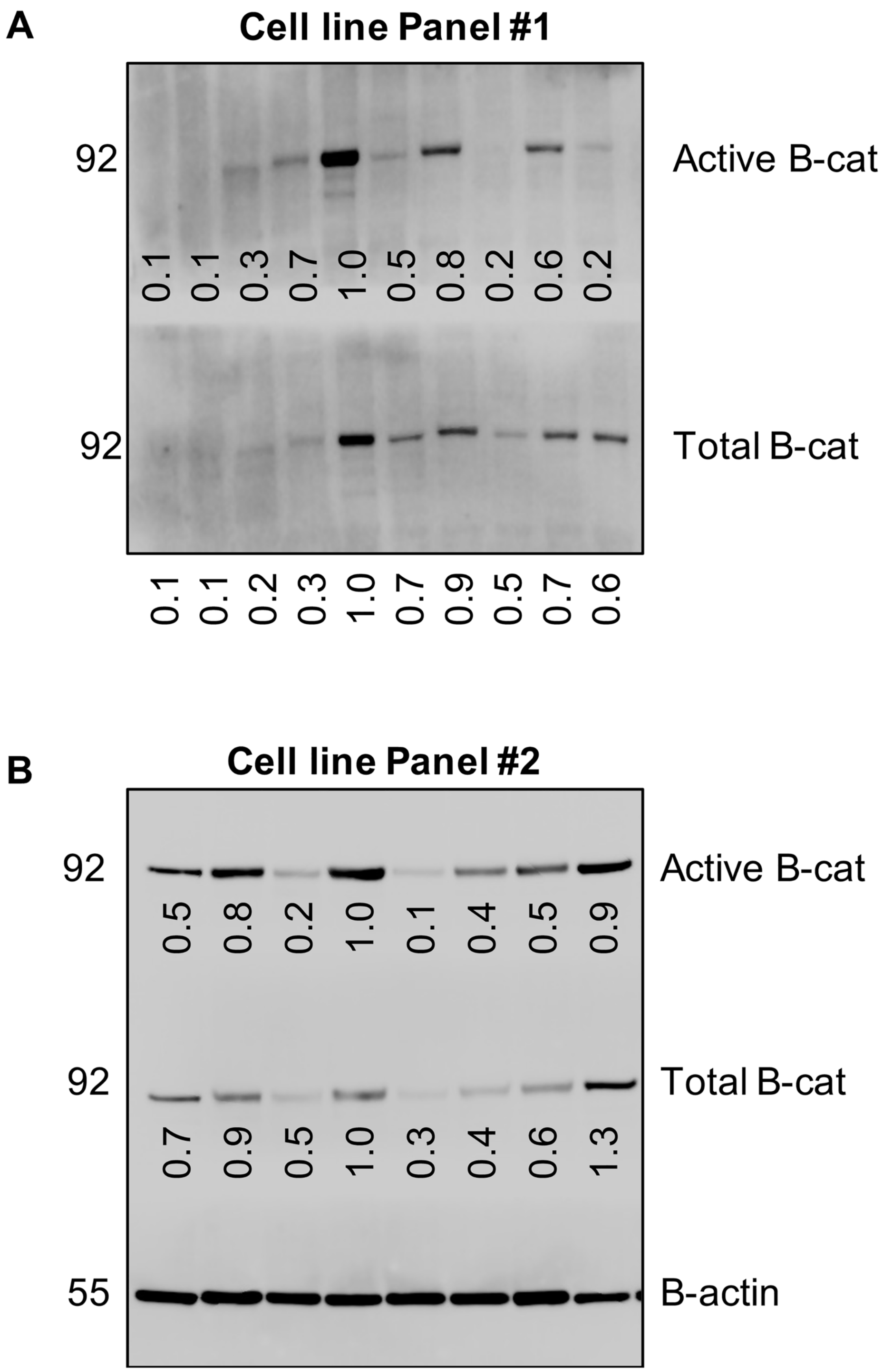

Supplementary Figure 4: Original western blots for Supplementary Figure 3. The blots evaluate the expression of Wnt pathway components in breast cancer cell lines. Total protein was extracted from two panels of different breast cell lines: human normal tissue (NT1, NT2, NT3), non-cancer mammary epithelial cell line (MCF10A and MCF12F), hormone receptor positive breast cancer cells (MCF-7, T-47D), triple-negative breast cancer cell lines (MDA-MB-231, MDA-MB-468 and BT-549), and patient-derived xenograft cell line (TX-BR-100). Protein expression patterns of endogenous active β- catenin, total β-catenin and β-actin were analysed by western blotting in breast cells lines as described above.

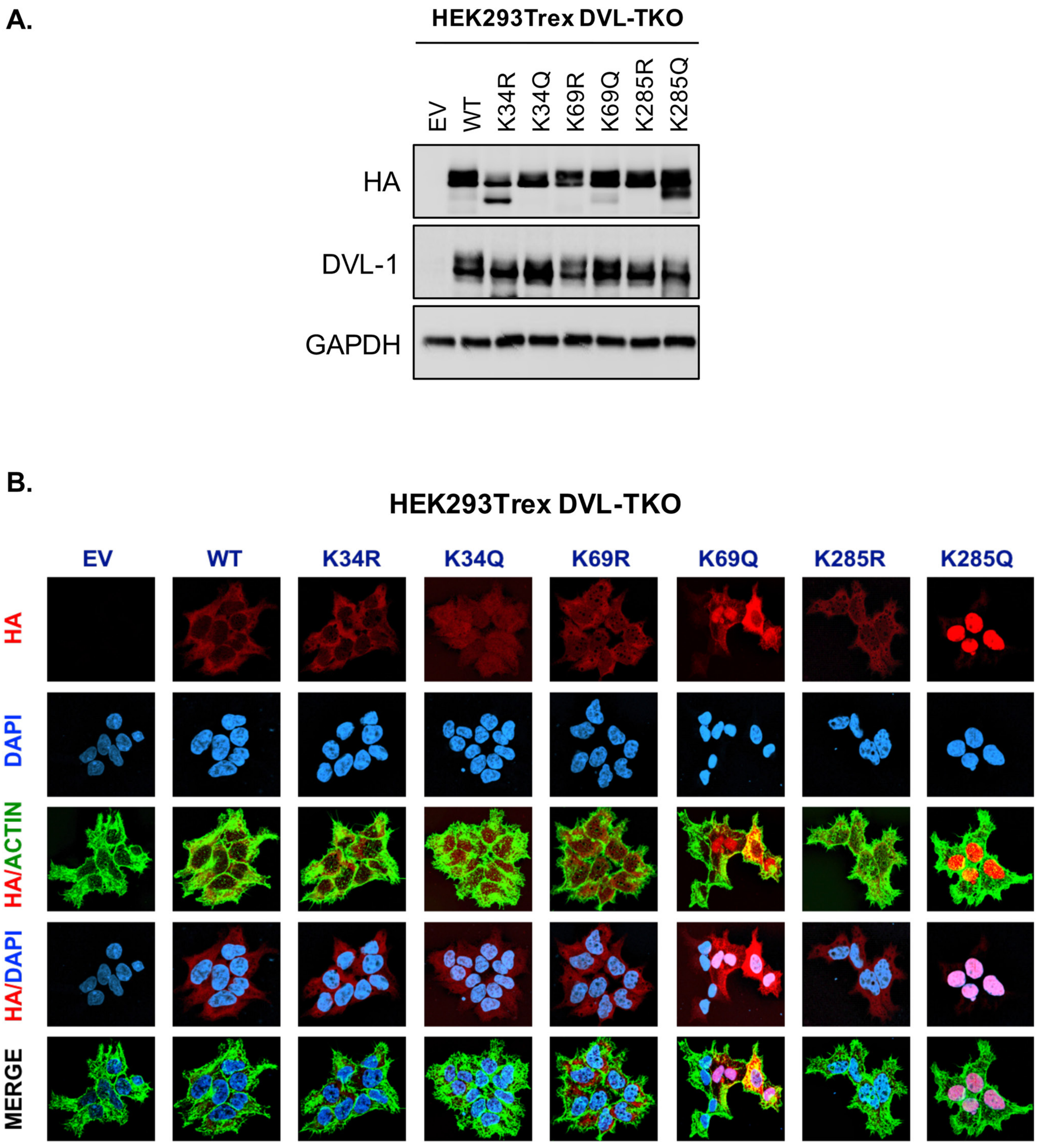

Supplementary Figure 5: Conserved lysine residues on the DIX and PDZ domains of human DVL-1 are critical for its subcellular localization. (**A**) Western blot analysis of lysates from HEK293Trex-DVL1/DVL2/DVL3 triple-knockout (DVL TKO) cells stably expressing empty vector (EV), N-terminal HA-epitope tagged DVL-1 wild type (WT), HA-tagged deacetylation mutants (K to R), HA-tagged acetylation mutants (K to Q) on highly conserved lysine residues namely, K34, K69 and K285, probed with antibody as indicated. (**B**) Immunofluorescence staining of empty vector (EV), N-HA-tagged DVL-1 (WT), K34R mutant (N-HA-K34R), K34Q mutant (N-HA-K34Q), K69R mutant (N-HA-K69R), K69Q mutant (N-HA-K69Q), K285R mutant (N-HA-K285R), K285Q mutants (N-HA-K285Q). Merge of N-HA-DVL-1 (red) and nuclear staining (blue) proteins is shown as HA/DAPI for each of the mutant. Merge of actin (green) and N-HA-DVL-1 (red) proteins is shown as HA/Actin for each of the mutant.

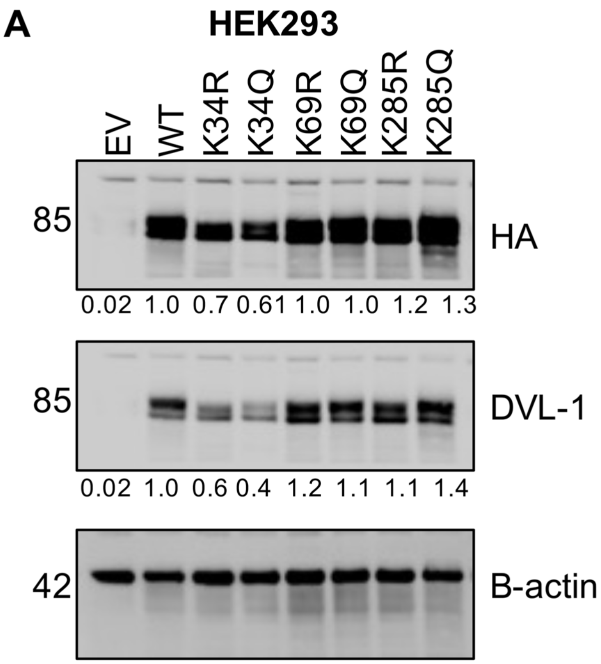

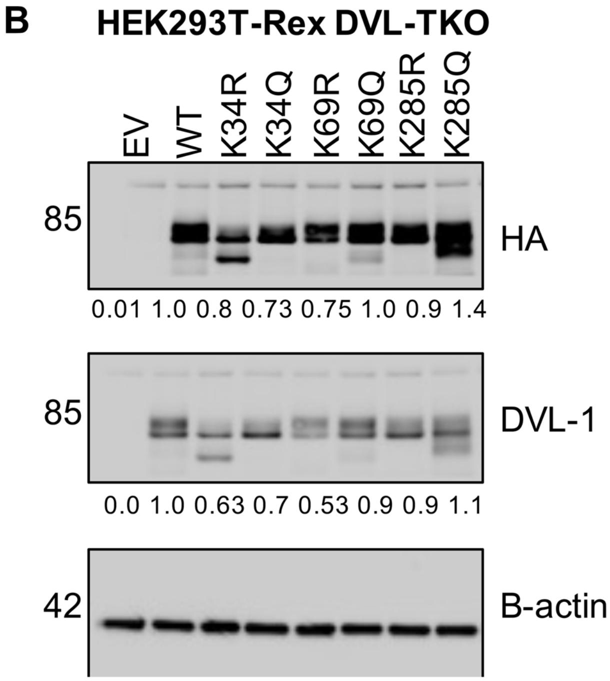

Supplementary Figure 6: Original western blots for Figure 2. Uncropped western blots of lysates from (**A**) HEK293 and (**B**) HEK293Trex-DVL1/DVL2/DVL3 triple-knockout (DVL TKO) cells stably expressing empty vector (EV), N-terminal HA-epitope tagged DVL-1 wild type (WT), HA-tagged deacetylation mutants (K to R), HA-tagged acetylation mutants (K to Q) on highly conserved lysine residues namely, K34, K69 and K285, probed with antibody as indicated. (**C**) Original western blots for Co-IP experiments shown in main Figure 2C–2D.

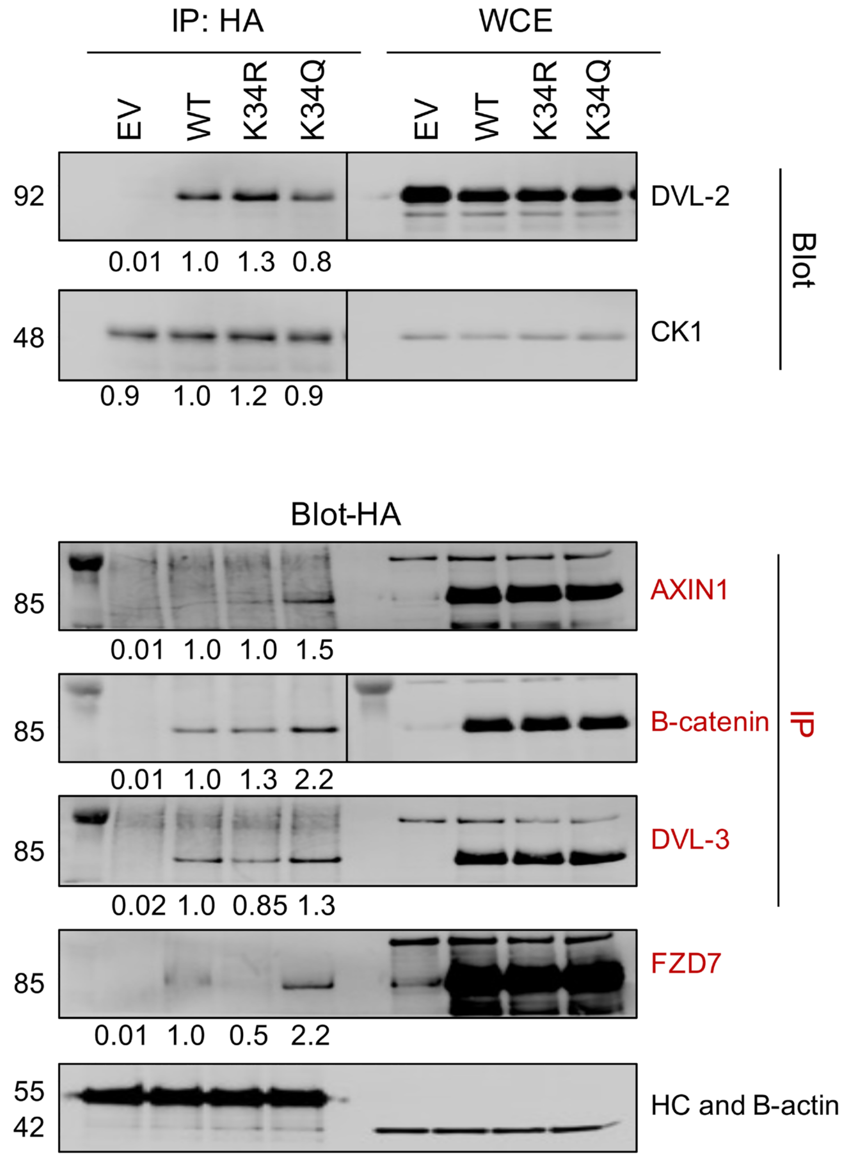

Supplementary Figure 7: Original western blots for Figure 2C–2D. Uncropped western blots for Co-IP experiments shown in main Figure 2C–2D.

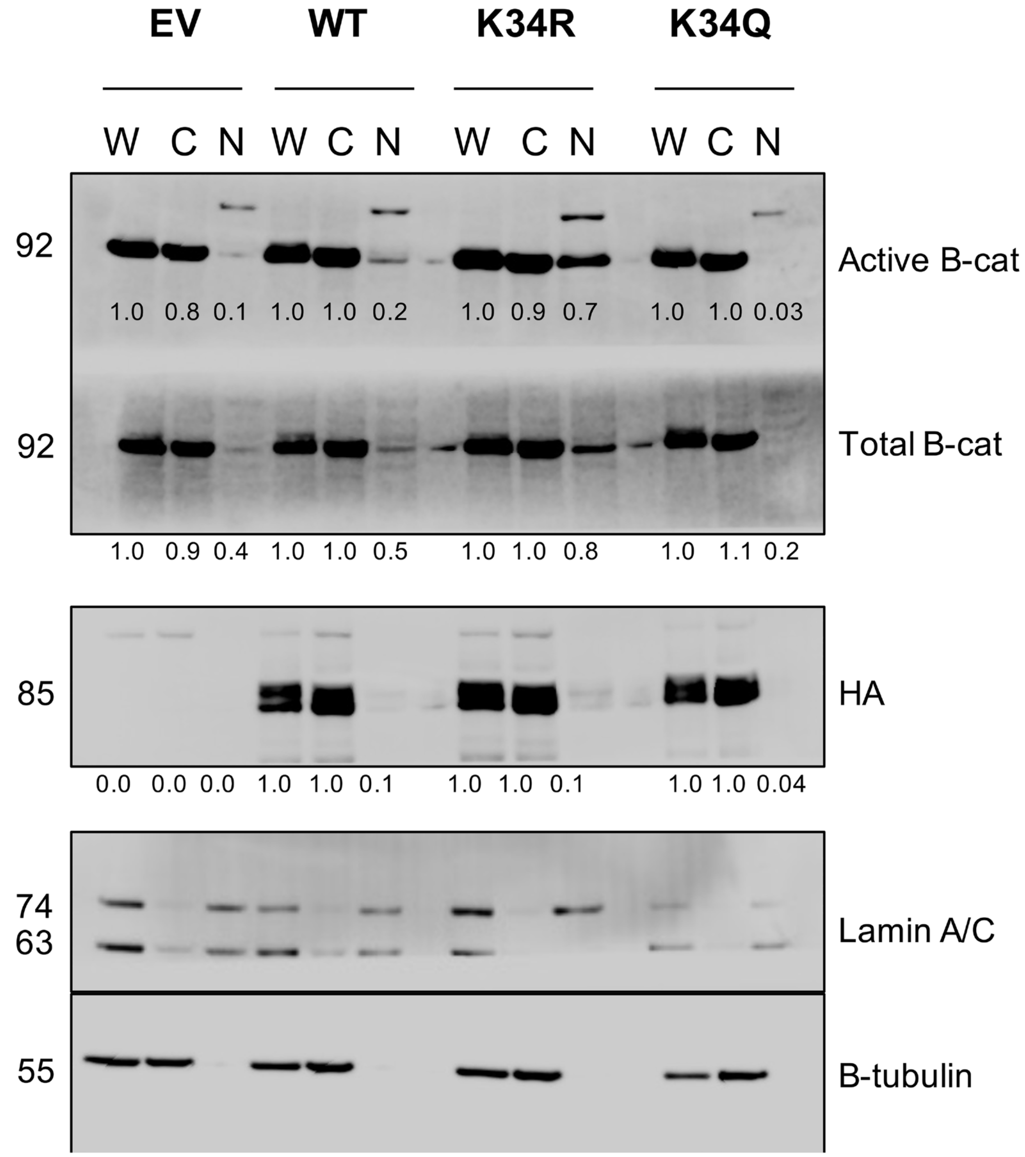

Supplementary Figure 8: Original western blots for Figure 2. Original blots for whole (W), cytoplasmic (C) and nuclear (N) extracts from HEK293 cells stably expressing EV, WT-DVL1, K34R, and K34Q were analyzed using Western blots. The blots were probed with antibodies against active β-catenin, total β-catenin, and HA-tag. Lamin was used as a control for nuclear extract and β-tubulin was used as a control for cytosolic proteins.

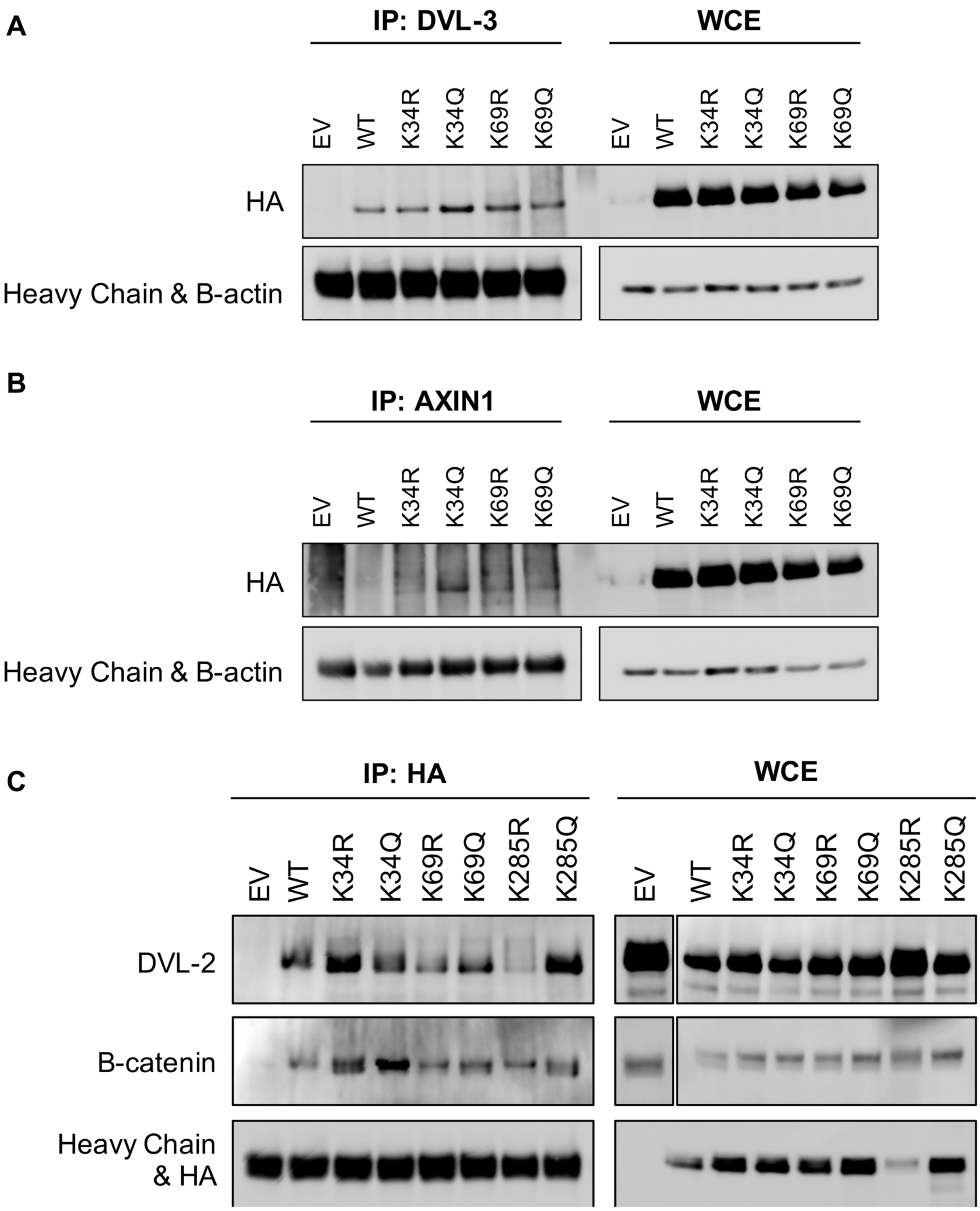

Supplementary Figure 9: Consequence of acetylation on conserved lysine residues and protein binding ability of DVL-1 protein. (**A**) DVL-1 differentially co-immunoprecipitates with multiple binding partners in K34, K69, K285 modified-state in HEK293 cells. Several binding partners such as DVL2, CK1e, DVL3, Axin1, and B- catenin were tested. Immunoprecipitation was performed using antibody against (A and **B**) protein binding partners or (**C**) with HA-tag antibody. Empty vector (EV) was used as a negative control, IgG heavy chain (IgG Hc) was blotted for as a control for equal antibody loading for immunoprecipitation. β-actin was included as a loading control for whole cell extract (WCE).

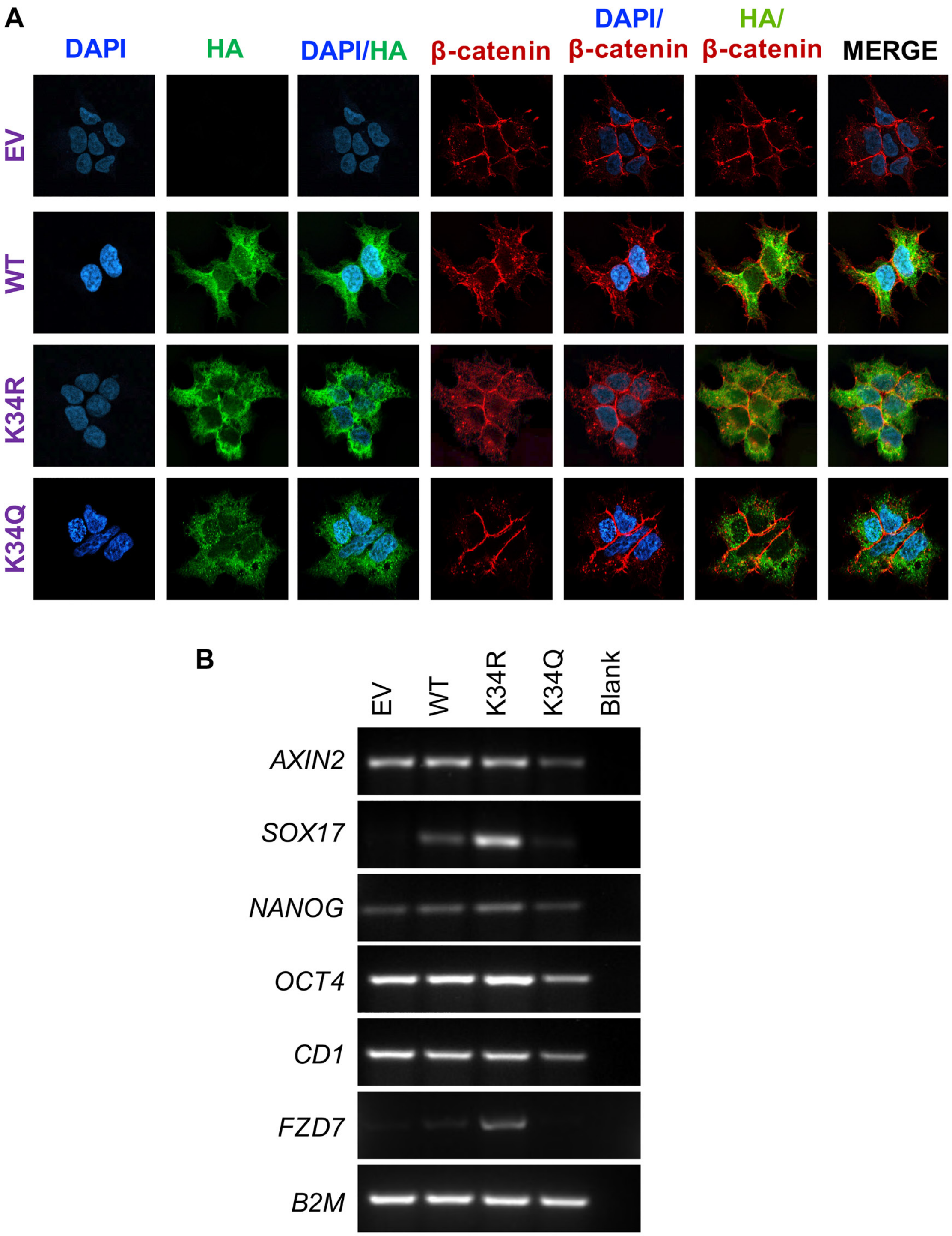

Supplementary Figure 10: Conserved K34 residue of DVL-1 disrupts nuclear localization of β-catenin, impairing downstream Wnt target gene expression. (**A**) Immunofluorescence staining of empty vector (EV), N-HA-tagged DVL-1 (WT), K34R mutant (N- HA-K34R), K34Q mutant (N-HA-K34Q) proteins in stably expressing HEK293 was conducted to analyse relative localization of β-catenin. Merge of nuclear staining (DAPI, blue), N-HA-DVL-1 (green), β-catenin (red) proteins is shown as DAPI/HA and DAPI/β-catenin for each of the mutant. Merge of β-catenin (red) and N-HA-DVL-1 (green) proteins is shown as β-catenin/HA for each of the mutant. (**B**) mRNA expression of WNT target genes such as *AXIN1, AXIN2, SOX17, NANOG, OCT4, FXD7, CYCLIN D1* and *B2M* (as loading control) was determined by end-point PCR in HEK293 cells stably expressing EV, WT-DVL1, K34R, and K34Q mutants.

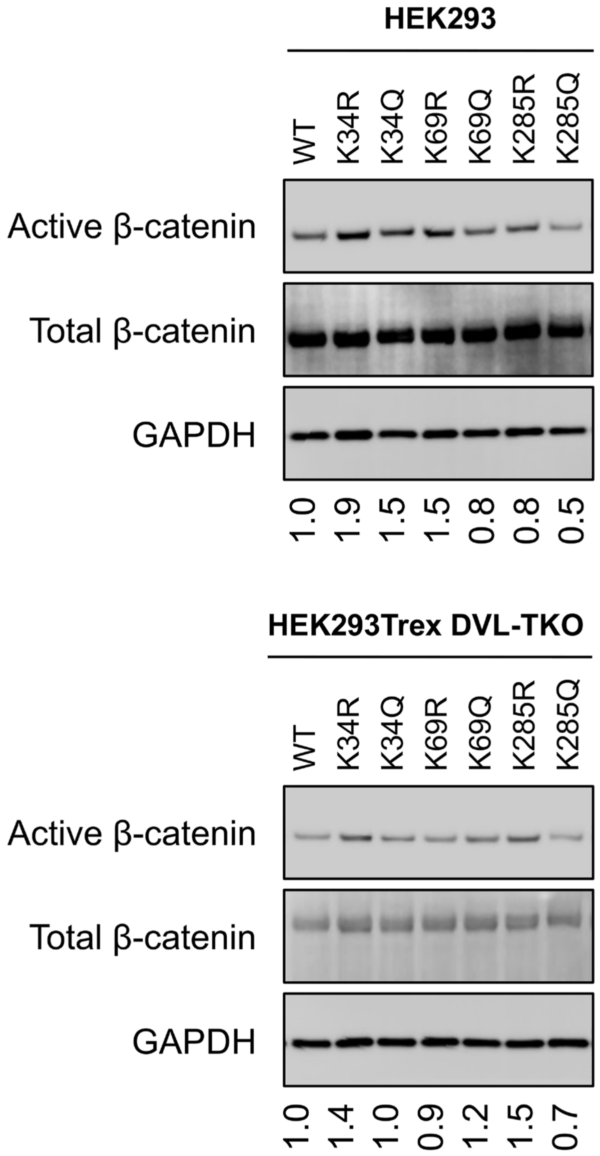

Supplementary Figure 11: Effect of DVL-1 mutants on β-catenin levels in HEK293 and HEK293Trex- DVL1/DVL2/DVL3 triple-knockout (DVL TKO) cells.

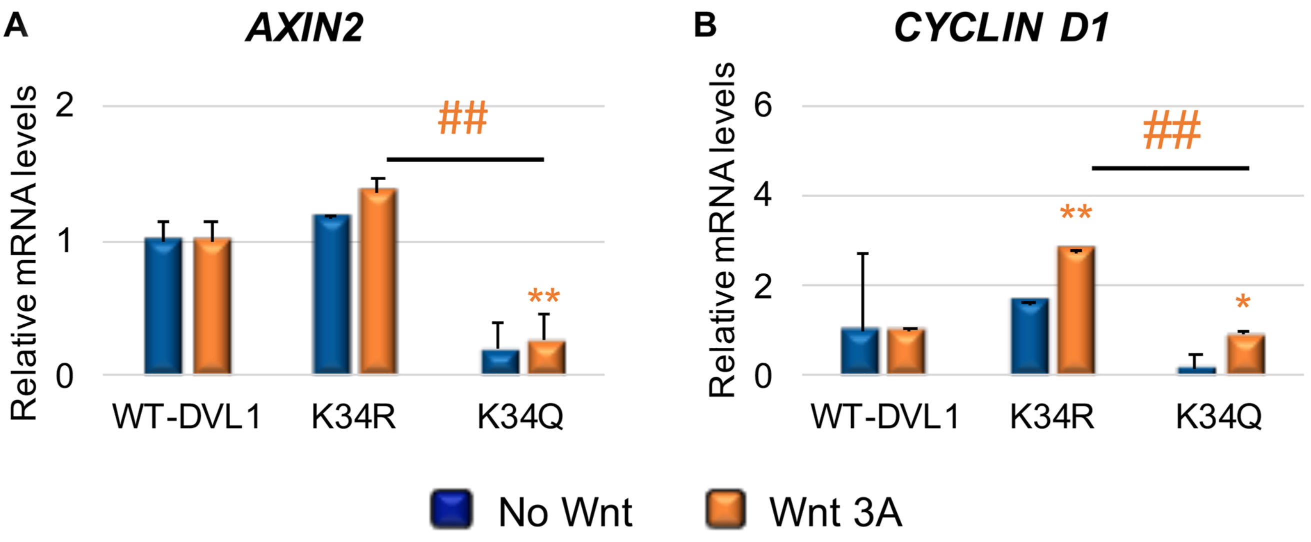

Supplementary Figure 12: Effect of Wnt3a stimulation on Wnt target genes (**A**) Axin2 and (**B**) CyclinD1 in WT versus K34 mutants in HEK293 cells. All results are from expressed as mean ± SEM and considered significant at $^{*/\#}p < 0.05$, $^{**/\#\#}p < 0.01$ and $^{***/\#\#\#}p < 0.001$. The sign "*" represents significant change between WT and mutants, while "#" represents significant change in R and Q mutants, with or without Wnt3a treatment.

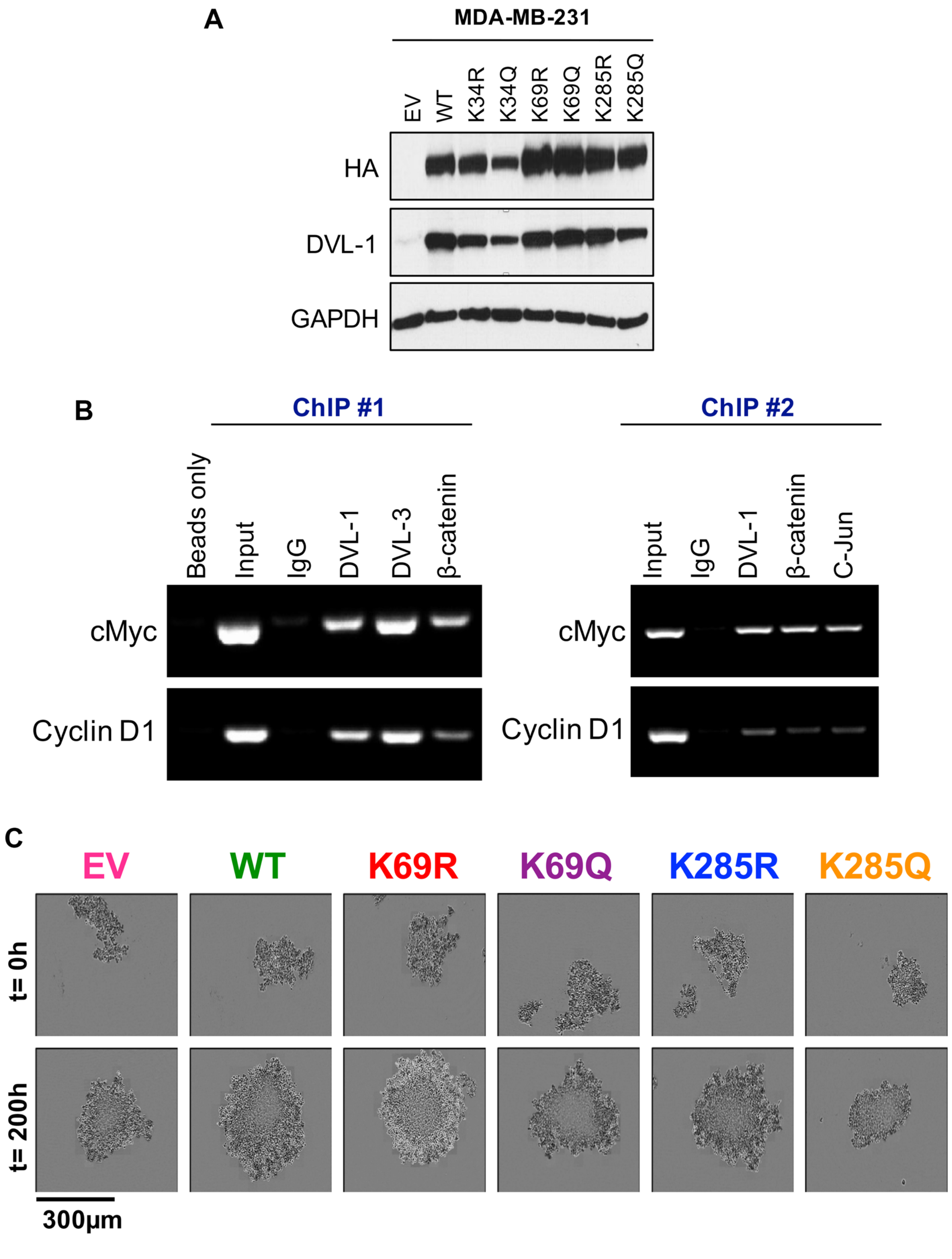

Supplementary Figure 13: Expression of DVL-1 point mutants in breast cell lines. (**A**) Stable expression of empty vector (EV), N-terminal HA-epitope tagged DVL-1 wild type (WT), HA-tagged deacetylation mutants (K to R), HA-tagged acetylation mutants (K to Q) on conserved lysine residues, K34, K69 and K285 in MDA-MB-231 cells. (**B**) Occupancy of controls such as DVL-3, β-catenin, and cJun along with DVL-1 at Wnt target *CMYC* and *CYCLIN D1* genes were analyzed by end-point PCR. ChIP experiments were performed in MDA-MB-231 cells. (**C**) Spheroid formation assay was conducted in MDA-MB-231 cell lines stably expressing DVL-1 mutants. The pictures represent spheroid area at t = 0 h and 200 h.

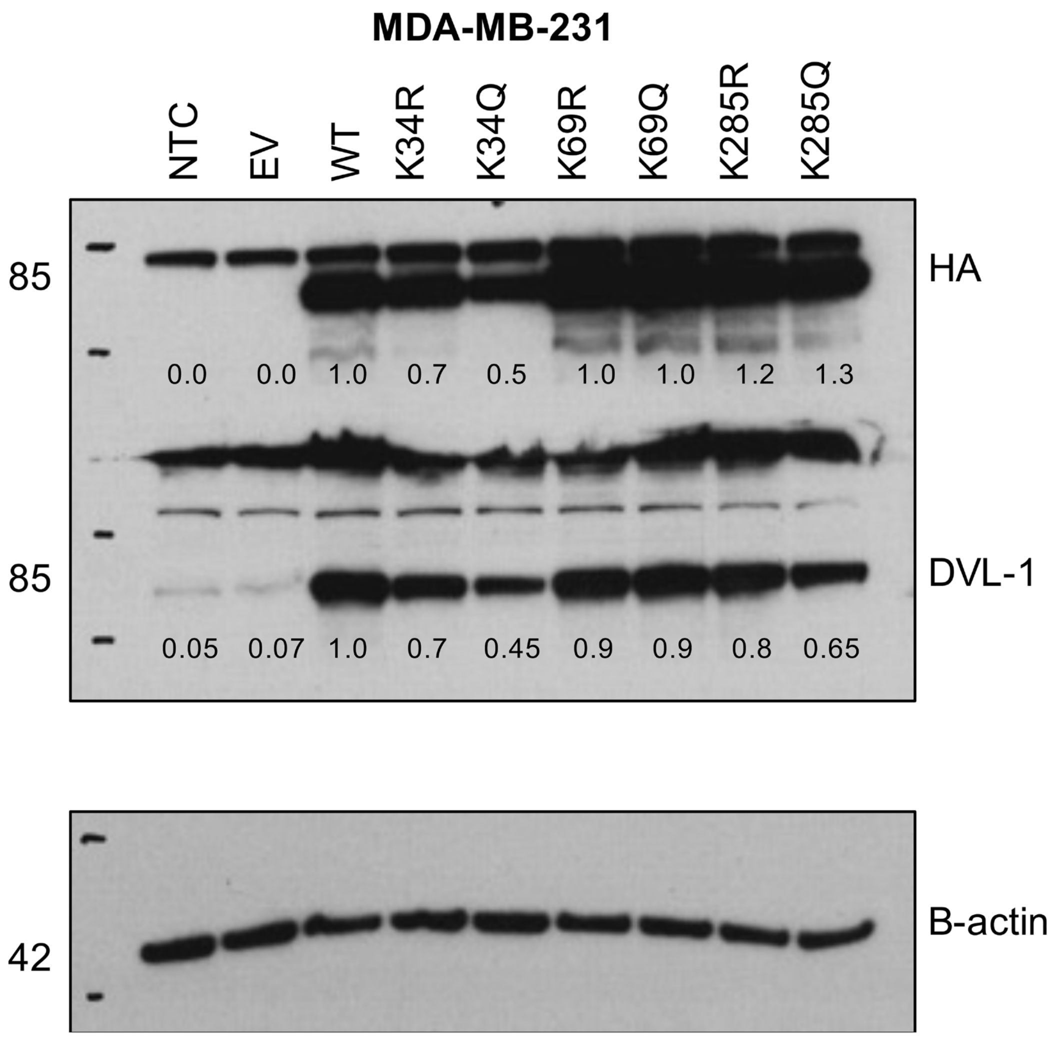

Supplementary Figure 14: Expression for DVL-1 mutants. Stable expression of empty vector (EV), N-terminal HA-epitope tagged DVL-1 wild type (WT), HA-tagged deacetylation mutants (K to R), HA-tagged acetylation mutants (K to Q) on conserved lysine residues, K34, K69 and K285 in MDA-MB-231 cells.

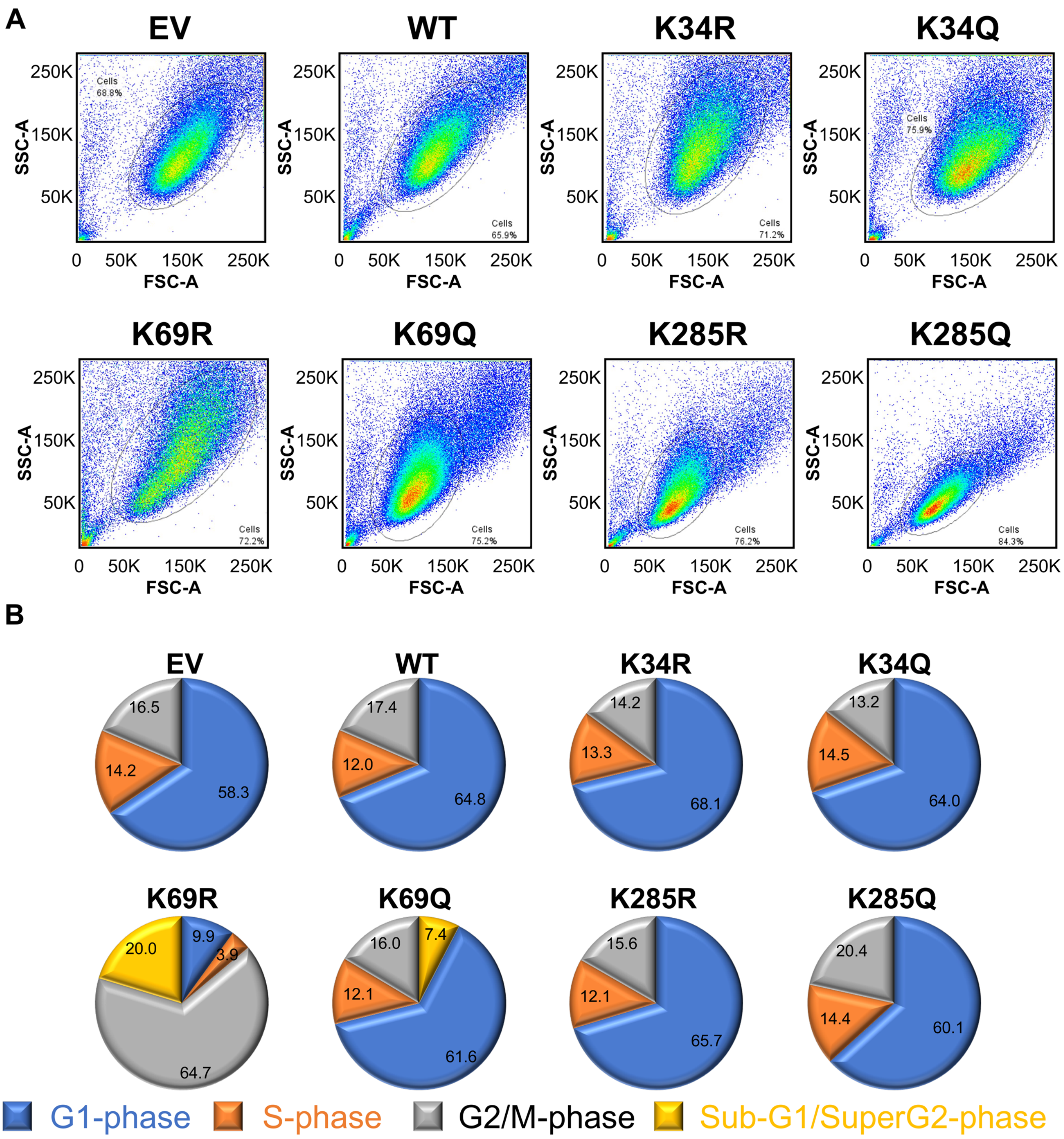

Supplementary Figure 15: DVL-1 acetylation on conserved lysines regulate cell cycle stages in breast cancer cells. (**A**) The effect of DVL-1 mutants was analyzed in a cell cycle assay using a Vybrant cell violet dye from Invitrogen followed by flow cytometry. The live cells are represented in the SSC versus FSC plots. (**B**) Pie chart representing different cell cycle stages (SubG1, G1, S, G2/M, and Super-G2) with DVL-1 mutations.

Forward ChIP primer
Reverse ChIP primer
TCF4 binding site (at 90% threshold)

>CMYC 5’ upstream region (0.7kb upstream of TSS)
cctggaacaggcagacacatctcagggctaaacagacgcctcccgcacggggccccacggaagcctga
gcaggcggggcaggaggggcggtatctgctgctttggcagcaaattgggggactcagtctgggtggaaggt
atccaatccagatagctgtgcatacataatgcataatacatgactcccccaacaaatgcaatgggagtttatt
cataacgcgctctccaagtatacgtggcaatgcgttgctgggttattttaatcattctaggcat**cgttttcctcctt**
atgcctctatcattcctccctatctacactaacatcccacgctctgaacgcgcgcccattaatacccttctttcctc
cactctccctgggactcttgatcaaagcgcggccctttccccagcctt**agcgaggcgccctgcagcctggt**
acgcgcgtggcgtggcggtgggcgcgcagtgcgttctcggtgtggag**ggcagctgtt**ccgcctgcgatgatt
tatactcacaggacaaggatgcggtttgtcaaacagtactgctacggaggagcagcagagaaagggagag
ggtttgagagggagcaaaagaaaatggtaggcgcgcgtagttaattcatgcggctctcttactctgtttacatcc
tagagctagagtgctcggctgcccggctgagtctcctccccaccttccccaccctccccaccctccccataag
cgcccctcccgggttcccaaagcagagggcgtgggggaaaagaaaaaagatcctctctcgctaatctccgc
ccaccggccctttataatgcgagggtctggacggctgaggacccccgagctgtgctgctcgcggccgccac
cgccgggccccggccgtccctggctcccctcctgcctcgagaagggcagggcttctcagaggcttggcggg
aaaaagaacggagggagggatcgcgctgagtataaaagccggttttcggggctttatct

>CD1 5’ upstream region (0.3kb upstream of TSS)
ggggactaatatttccagcaatttaatttctttttaattaaaaaaaatgagtcagaatggagatcactgtttctcag
ctttccattcagaggtgtgtttctcccggttaaattgccggcacgggaagggagggggtgcagttggggacccc
cgcaaggaccgactggtcaaggtaggaaggcagcccgaagagtctccaggctagaaggacaagatgaa
ggaaatgctggccaccatcttgggctgctgctggaattttcgggcatttattttattttatttttgagcgagcgcatg
ctaagctgaaatccctttaacttttagggttaccccccttgggcatttgcaacgacgcccctgtgcgccggaatga
aacttgcacaggggttgtgtgcccggtcctccccgtccttgcatgctaaattagttcttgcaatttacacgtgttaat
gaaaatgaaagaagatgcagtcgctgagattctttggccgtctgtccgcccgtgggtgccctcgtggcgttcttg
gaaatgcgcccattctgccggcttggatatggggtgtcgccgcgccccagtcaccccttctcgtggtctcccca
ggctgcgtgtggcctgccggccttcctagttgtcccctactgcagagccacctccacctcacccccctaaatccc
gggggacccactcgaggcggacggggccccctgcacccctcttccctggcggggagaaaggctgcagcg
gggcgatttgcatttctatgaaaaccggactacaggggcaactccgccgcagggcaggcgcggcgcctca
gggatggcttttgggctctgcccctcgctgctcccggcgtttggcgcccgcgccccctcccccctgcgcccgccc
ccgccccccctcc**cgctcccattctctgccggg**ctttgatctttgcttaacaacagtaacgtcacacggactac
aggggagttttgttgaagttgcaaagtcctggagcctccagagggctgtcggcgcagtagcagcgagcagc
agagtccgcacgctccggcgagggggcag**aagagcgcgagggagcgcgg**ggcagcagaagcgaga
gccgagcgcggacccagccaggacccacagccctc**cccagctgccc**aggaagagccccagcc

Supplementary Figure 16: Putative TCF binding sites on 5′ upstream region of cmyc and CyclinD1 (CD1).
